# Reticular Basement Membrane Thickness Is Associated with Growth- and Fibrosis-Promoting Airway Transcriptome Profile-Study in Asthma Patients

**DOI:** 10.3390/ijms22030998

**Published:** 2021-01-20

**Authors:** Stanislawa Bazan-Socha, Sylwia Buregwa-Czuma, Bogdan Jakiela, Lech Zareba, Izabela Zawlik, Aleksander Myszka, Jerzy Soja, Krzysztof Okon, Jacek Zarychta, Paweł Kozlik, Sylwia Dziedzina, Agnieszka Padjas, Krzysztof Wojcik, Michal Kepski, Jan G. Bazan

**Affiliations:** 1 Department of Internal Medicine, Jagiellonian University Medical College, 31-066 Krakow, Poland; bogumil.j@poczta.fm (B.J.); jerzysoja1@gmail.com (J.S.); jzar@mp.pl (J.Z.); pawelkozlik89@gmail.com (P.K.); sylwiaz49@poczta.fm (S.D.); agnieszkapadjas@gmail.com (A.P.); krzysztof.wojcik@uj.edu.pl (K.W.); 2 College of Natural Sciences, Institute of Computer Science, University of Rzeszów, Pigonia 1, 35-310 Rzeszów, Poland; sczuma@ur.edu.pl (S.B.-C.); lzareba@ur.edu.pl (L.Z.); mkepski@ur.edu.pl (M.K.); jan.g.bazan@gmail.com (J.G.B.); 3 Centre for Innovative Research in Medical and Natural Sciences, Institute of Medical Sciences, Medical College, University of Rzeszów, Kopisto 2a, 35-959 Rzeszów, Poland; izazawlik@yahoo.com (I.Z.); amyszka@univ.rzeszow.pl (A.M.); 4 Department of Pathology, Jagiellonian University Medical College, Grzegorzecka 16, 31-531 Krakow, Poland; k.okon@uj.edu.pl; 5 Pulmonary Hospital, Gladkie 1, 34-500 Zakopane, Poland

**Keywords:** asthma, airway remodeling, gene expression, bronchial epithelium

## Abstract

Airway remodeling in asthma is characterized by reticular basement membrane (RBM) thickening, likely related to epithelial structural and functional changes. Gene expression profiling of the airway epithelium might identify genes involved in bronchial structural alterations. We analyzed bronchial wall geometry (computed tomography (CT)), RBM thickness (histology), and the bronchial epithelium transcriptome profile (gene expression array) in moderate to severe persistent (*n* = 21) vs. no persistent (*n* = 19) airflow limitation asthmatics. RBM thickness was similar in the two studied subgroups. Among the genes associated with increased RBM thickness, the most essential were those engaged in cell activation, proliferation, and growth (e.g., *CDK20*, *TACC2*, *ORC5*, and *NEK5*) and inhibiting apoptosis (e.g., higher mRNA expression of *RFN34*, *BIRC3*, *NAA16*, and lower of *RNF13*, *MRPL37*, *CACNA1G*). Additionally, RBM thickness correlated with the expression of genes encoding extracellular matrix (ECM) components (*LAMA3*, *USH2A*), involved in ECM remodeling (*LTBP1*), neovascularization (*FGD5*, *HPRT1*), nerve functioning (*TPH1*, *PCDHGC4*), oxidative stress adaptation (*RIT1*, *HSP90AB1*), epigenetic modifications (*OLMALINC*, *DNMT3A*), and the innate immune response (*STAP1*, *OAS2*). Cluster analysis revealed that genes linked with RBM thickness were also related to thicker bronchial walls in CT. Our study suggests that the pro-fibrotic profile in the airway epithelial cell transcriptome is associated with a thicker RBM, and thus, may contribute to asthma airway remodeling.

## 1. Introduction

Asthma is an inflammatory disease of the airways that may result from exposure to inhaled allergens or other environmental agents, rendering genetically susceptible individuals to aberrant activation and prolonged immune responses [1]. Airway remodeling is a pathological feature of persistent asthma and contributes to the clinical manifestations of the disease. It refers to the bronchial wall’s structural changes caused by chronic inflammation, repeated cycles of injury, and repair [2]. In asthma, airway remodeling is characterized by modifications in the epithelium and subepithelial fibrosis, including thickening of the basement membrane and deposition of extracellular matrix (ECM) proteins in the submucosa [3,4]. Thickening of the basement membrane occurs mainly in the lamina reticularis layer, the so-called reticular basement membrane (RBM), which is localized beneath the basal lamina [3].

Although host factors that make asthma subjects susceptible to airway structural changes are complex and poorly understood, multiple studies support the concept of airway epithelial barrier dysfunction as being critical for this process [1,5]. The airway epithelium is continuously exposed to airborne particles and infectious agents and represents the airways’ frontline barrier between the host and environment. Furthermore, it plays a vital role in the innate immune defense and mucociliary clearance. As a source of cytokines and growth factors, the airway epithelium regulates other cell functions [5,6]; for example, it stimulates lung fibroblasts to produce ECM components, such as collagens and fibronectin, and pro-fibrotic transforming growth factor (TGF)-β [1]. Moreover, known environmental risk factors, such as cigarette smoke, biomass fuel particles, and respiratory viral infections, may stimulate the production of ECM proteins by the airway epithelium, fibroblasts, and smooth muscle cells [1].

Under physiological conditions, the epithelium’s integrity is preserved by tight junctions, limiting the passage of macromolecules to the submucosa. Exposure to allergens, pathogens, or air pollutants can cause cleavage of tight junctions, increasing epithelial permeability, which likely further enhances persistent airway inflammation in asthma [7]. In predisposed individuals, such environmental injury may also induce airway epithelial cell apoptosis, accompanied by the secretion of paracrine factors that initiate the regenerative process in the tissue, leading to the aberrant repair and subsequent structural changes of the airway [1]. 

Genome-wide association studies have identified several susceptibility genes associated with epithelial barrier function, differentiation, and homeostasis, including genes encoding cadherin family proteins and those related to mucus production [7]. However, genetic factors’ exact contribution to epithelial barrier defects, asthma development, progression, and airway remodeling are not entirely understood. Moreover, bronchial epithelial gene expression signatures and their linkage to the airway’s structural changes have not been previously studied. Therefore, by analyzing bronchial mucosa biopsies from asthma individuals, we investigated potential associations between the epithelial cell transcriptome and RBM thickness, as well as their linkage to asthma severity, inflammation patterns, persistent airflow limitation, and airway geometry parameters revealed by lung computed tomography (CT) scans. According to our knowledge, such an approach has not been previously implemented in asthma research.

## 2. Results

### 2.1. Patient Description and Basic Laboratory Tests

We analyzed 40 non-smoking moderate to severe asthma patients aged 30–65 years, with at least a 3-year history of confirmed asthma and no exacerbation within at least six months before the enrollment. About half of the patients (*n* = 21, 52.5%) had persistent airflow limitation, while the remaining (*n* = 19, 47.5%) had normal spirometry before or after bronchodilator. Persistent airflow limitation was defined as a forced expiratory volume in 1 s (FEV_1_)/vital capacity (VC) index below 0.7 or FEV_1_ lower than 0.8 of predicted value after bronchodilator.

Demographics and clinical characteristics of patients studied are provided in Table 1.

Both subgroups did not differ in the demographic parameters or asthma severity and duration. Patients with persistent airflow limitation were characterized by higher blood and bronchoalveolar lavage (BAL) eosinophilia (Table 2). Intriguingly, they also had lower interleukin (IL)-6 concentrations in BAL.

### 2.2. Computed Tomography Imaging Shows Airway Wall Thickening in Asthma Patients with Persistent Airflow Limitation

**Figure 1:**
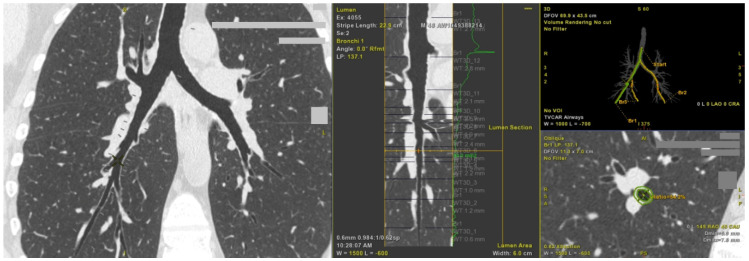
Lung computed tomography—automated measurements of airway geometry parameters in the right lower lobe basal posterior bronchus (RB10).

Figure 1 depicts how airway geometry parameters were automatically analyzed.

Table 3 presents cross-sectional geometry parameters of the right upper lobe apical segmental bronchus (RB1) and the right lower lobe basal posterior bronchus (RB10).

As expected, corresponding airway cross-sectional geometry parameters measured in RB1 and RB10 correlated well with each other; for wall area ratio (WAR) and wall thickness ratio (WTR), the correlation coefficients were the strongest (R = 0.53, *p* = 0.001, and R = 0.55, *p* < 0.001, respectively).

CT imaging revealed a higher RB1 WAR and WTR in subjects with persistent airflow limitation and a tendency towards an increase in those variables in RB10 (Table 3). That group of patients also had lower lumen and airway diameters and decreased lumen areas in RB10 than the remaining individuals (Table 3). Males were characterized by higher airway and lumen areas; however, WAR and WTR were not associated with gender. Additionally, lumen and wall diameters in RB10 remained in a weak positive relationship with height (R = 0.37, *p* = 0.02, and R = 0.32, *p* = 0.049, respectively).

WAR and WTR in RB1, but not in RB10, inversely correlated with the FEV_1_/VC index before bronchodilator (R = −0.34, *p* = 0.04, and R = −0.37, *p* = 0.03, respectively).

Additionally, RB1 wall thickness was positively related with peripheral blood and BAL eosinophilia (R = 0.45, *p* = 0.009, and R = 0.53, *p* = 0.001, respectively). A similar, although less strong association was documented between RB1 WAR or WTR and blood eosinophil count (R = 0.35, *p* = 0.04, and R = 0.36, *p* = 0.04, respectively).

### 2.3. Reticular Basement Membrane Thickness Is Not Increased in Asthma Patients with Persistent Airflow Limitation

**Figure 2:**
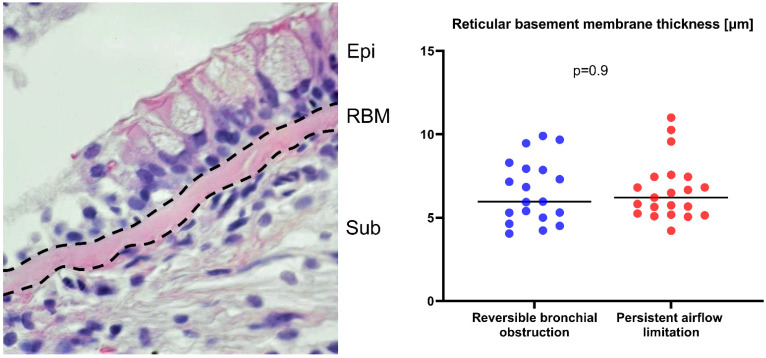
Histological examination of the bronchial mucosa section; abbreviations: Epi—epithelium, RBM—reticular basement membrane (shown by dashed lines), Sub—submucosa. The reticular basement thickness was similar in both asthma subgroups.

Surprisingly, RBM thickness was not significantly increased in asthma subjects with persistent airflow limitation (Figure 2).

RBM thickness was also not related to age, sex, asthma duration, and smoking history (data not shown). Likewise, there was no association with spirometry values, asthma severity, or symptom control scores. Among laboratory parameters, thickness of the RBM layer showed a moderate positive correlation with blood neutrophil count (R = 0.35, *p* = 0.02) and BAL periostin (R = 0.46, *p* = 0.04), albeit not with systemic biomarkers of type-2 inflammation (blood eosinophil count: R = −0.25, *p* = 0.09; serum periostin: R = −0.3, *p* = 0.2). Additionally, patients with a thicker RBM (median value as a cut-off point (6.08 µm)) were characterized by a higher odds ratio (3.95 (95%CI: 1.75–6.2), *p* < 0.001) of increased BAL periostin concentrations, defined as values above the cut-off point of 0.95 ng/mL.

RBM thickness was not related to lung CT airway geometry parameters, except for a weak positive relationship with RB10 wall thickness (R = 0.3, *p* = 0.04), after adjustment for confounders (β = 0.39 (95%CI: 0.2–0.6)).

### 2.4. Increased Reticular Basement Membrane Thickness Is Associated with Fibrosis- and Growth-Promoting Pattern of Gene Expression in Bronchial Epithelium

To search for unique airway transcriptome patterns associated with increased RBM thickness, we stratified asthma patients into those with increased vs. decreased RBM thickness, using the median value as a cut-off point (6.08 µm). This comparison revealed 51 genes, for which expression significantly differed between both analyzed subgroups. Among them, 17 genes differed at the significance level lower than 0.001. These genes are described in Table S1 (Supplementary Materials). The thicker RBM was linked with a gene expression profile promoting cell proliferation and growth (*CDK20* [8], *MIS12* [9], *PDS5B* [10]), regulating DNA transcription (*ZNF594* [11]), apoptosis (*RNF157* [12], *HTRA2* [13]), and cargo transporting (*SLC39A13* [14], *SLC16A6* [15], *IPO13* [16]), as well as related to the ECM components (*LAMA3* [17], *USH2A* [18]). Moreover, we documented higher mRNA levels of *CLEC9A* (C-type lectin domain containing 9A) [19], which encodes an endocytic receptor specialized in the uptake and processing of dead cell material, and *OAS2*, encoding 2′,5′-oligoadenylate synthase, engaged in the cell antiviral response [20]. Interestingly, we also identified differentially expressed genes involved in neuronal cell proliferation, growth, and function. For example, increased RBM thickness was associated with the lower expression of *BRINP2*, which inhibits neuronal cell proliferation (Table S1) [21].

Further correlation analyses identified several genes in which expression was positively (101 genes) or negatively (39 genes) associated with RBM thickness. Among them, 19 and nine, respectively, had particularly strong correlation coefficients (i.e., ≥ 0.6 or ≤ −0.6). This set of genes is listed in Table 4.

**Figure 3:**
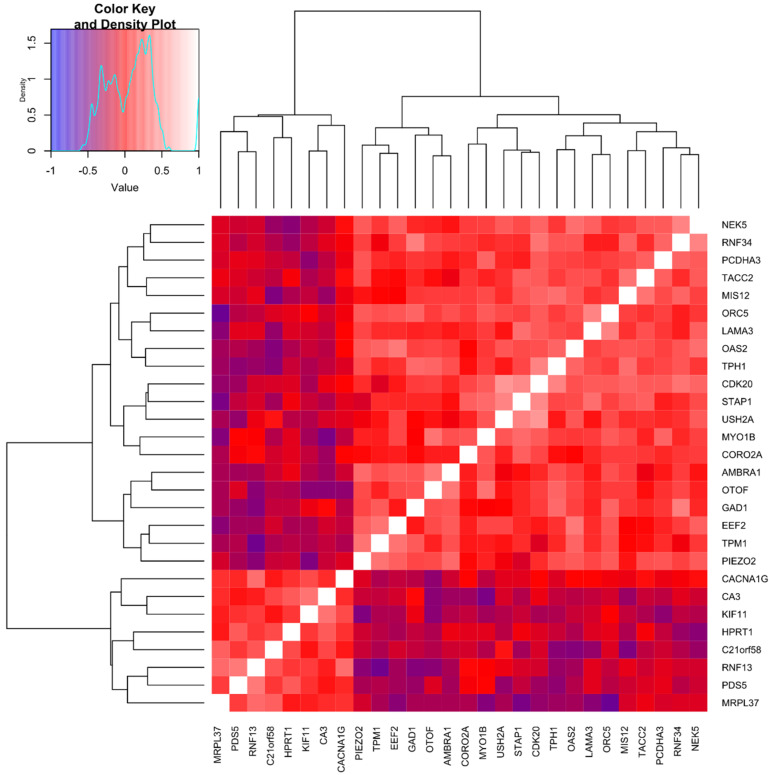
Spearman’s rank cross-correlation matrix and hierarchical clustering of 28 genes which expression correlated best with reticular basement membrane thickness.

In Figure 3, we present the cross-correlation matrix and hierarchical clustering (a heatmap) of 28 genes, for which expression correlated best with RBM thickness, i.e., with correlation coefficients ≥ 0.6 or ≤ −0.6. This Figure demonstrates which genes (among those 28) were associated well with each other. For example, some of the genes for which expression remained in a strong inverse relationship with RBM thickness, e.g., *MRPL37*, were also negatively associated with the mRNA level of genes promoting cell proliferation and growth, but determining RBM thickness positively (e.g., *ORC5*, *CDK20*, *EEF2*). On the other hand, a strong association was demonstrated between mRNA levels of *TPMI1* and *CDK20* or *TACC2* and *AMBRA1*, likely indicating similar regulatory mechanisms of their expressions.

The remaining genes significantly correlated with RBM thickness (i.e., with less strong coefficients) are listed in Supplementary Materials (Tables S2 and S3).

**Figure 4:**
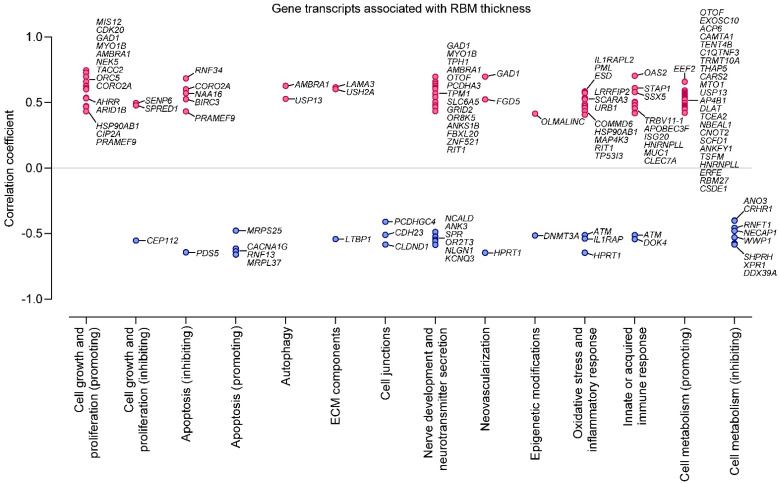
Bronchial epithelial cell transcriptome that significantly correlated with reticular basement membrane (RBM) thickness. For clarity, only genes with correlation coefficients lower than −0.4 and higher than 0.4 are shown. Genes were grouped into functional categories based on the gene literature and ontology data.

As expected, correlation analysis confirmed most of the genes differentially expressed in patients with higher RBM thickness (e.g., *LAMA3* [17], *USH2A* [18], *MIS12* [9], *CDK20* [8]). However, we identified other genes potentially contributing to RBM depositions, including *TACC2*, encoding centrosome- and microtubule-interacting protein [22], *NEK5* regulating centrosome integrity [24], *ORC5* modulating DNA replication [25], *AMBRA1* involved in autophagy, cell survival, and proliferation [26], as well as *MYO1B* and *CORO2A*, both controlling various cellular processes, such as cell cycle progression, signal transduction, cell motility, apoptosis, and gene expression [29,30]. Interestingly, many of the genes positively correlated with RBM thickness have anti-apoptotic functions (e.g., *RNF34* [35]), are involved in immune responses (*OAS2* [20], *STAP1* [37]), the cell contractile system (*TPM1*, *TPM2*, *MYO1B*, *CORO2A* [29,30,31]), neuronal growth and function (*PCDHA3* [50]), or have essential enzymatic activities (*GAD1* [27], *TPH1* [33], *EEF2* [36]). On the other hand, several genes inversely associated with the RBM thickness possess opposite biological functions, such as *PDS5* [10] which negatively regulates cell proliferation, and apoptosis promoting genes, including *RNF13* [43], *MRPL37* [44,45], and *CACNA1G* [46]. Overall, most of the genes associated with RBM thickness could be assigned to ontology groups linked with a cell growth-promoting phenotype (e.g., cell proliferation and metabolism, anti-apoptotic) and fibrosis/remodeling-promoting phenotype (e.g., ECM components and proteins regulating neovascularization) as summarized in Figure 4. This Figure was prepared based on gene ontology terms presented in Tables S4–S6 in Supplementary Materials and the literature data.

**Figure 5:**
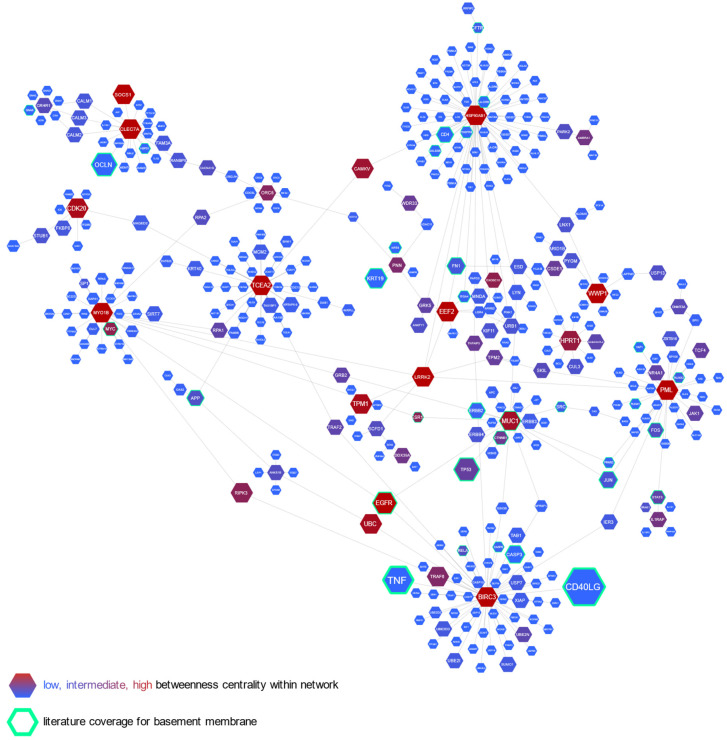
Graph of lung-specific interactions of genes for which expression correlated with reticular basement membrane (RBM) thickness. Interactions retrieved from the DifferentialNet database. Literature coverage regards the keyword “basement membrane”.

In contrast, in Figure 5, we present a network of lung-specific interactions of those genes. As has been shown, the processes modulated by the indicated genes have a wide range of interdependencies. We identified several hubs with interlinking genes, associated with basement membrane structure or functions, either directly or through first-degree interactors. By betweenness centrality within the network, *HSP90AB1*, encoding multifunctional chaperon protein, *BIRC3* regulating apoptosis, *HPRT1* referred to the purine salvage pathway uric acid production, and *TCEA2*, involved in RNA polymerase II functioning, formed the largest hubs. The lower nodes referred to the *PML* gene, modulating DNA transcription, cell apoptosis, and senescence; *MYOB1*, engaged in actin filament-based movement, and *MUC1* or *CLEC7A* encoding membrane receptors.

### 2.5. Cluster Analysis Integrating Molecular and Remodeling Data Reveals Subgroups of Asthma Patients with Unique Bronchial Epithelial Gene Expression Patterns

A cluster analysis based on epithelial expression levels of genes that correlated best with RBM thickness (28 genes included) and clinical data revealed three different clusters of patients. Clusters one (*n* = 11) and two (*n* = 10) were characterized by altered expression of six genes related to cell growth and proliferation or possessing anti-apoptotic properties (i.e., increased expression of *CDK20*, *RNF34*, *GAD1*, *PDS5* and lower of *RNF13* and *MRPL37*), involved in ECM composition (*LAMA3*), innate immune response (*OAS2*), contractile system (*TPMI1*, *PIEZO2*), and *TPH1*, as compared to cluster three (*n* = 19). Cluster two had the highest expression of additional genes engaged in ECM structure (*USH2A*) and cell growth (*EEF2*, *TACC2*, *NEK5*), together with the most significant decline in *HPRT*, compared with the two remaining collections. Cluster two has also been referred to as having increased expression of the other two genes involved in cell growth (*ORC5*, *AMBRA1*) and *STAP1* and *OTOF*, compared to cluster three.

Interestingly, clusters one and two had thicker RBMs in comparison to cluster three (*p* < 0.001, both), while cluster two referred to the highest values of RB1 and RB10 wall thickness, as compared to the remaining patient collections (*p* < 0.008 and *p* = 0.049, respectively). Cluster one had a higher BAL periostin than cluster three (*p* = 0.01). Demographic and clinical parameters and spirometry values did not differ among the three separated sets of patients.

## 3. Discussion

In this study, we compared several airway remodeling indices at the large bronchi and peripheral level, including CT airway cross-sectional geometry and histological examination of the mucosa (RBM thickness) in asthma subjects, with a particular focus on individuals with persistent airflow limitation vs. those with normal spirometry after bronchodilator. We also investigated the associations between the bronchial epithelial cell transcriptome and airway structural parameters. We have demonstrated that altered gene expression in the bronchial epithelium may determine RBM thickness, which was surprisingly weakly related to other airway remodeling markers. First of all, the RBM layer was not increased in patients with persistent airflow limitation. It either did not correlate with CT bronchial wall remodeling indices, except for a weak relationship with RB10 wall thickness. On the other hand, we have shown a clear association between the degree of airflow limitation and airway remodeling indices in lung imaging, particularly within the RB1. Therefore, we may speculate that RBM thickening likely progresses independently of the parameters linked with airway geometry and function, such as the rate of the decrease in spirometry values and the altered bronchial wall in CT scans.

Interestingly, airway and systemic biomarkers of type-2 inflammation (e.g., blood and BAL eosinophilia) were higher in patients with persistent airflow limitation and those with thicker walls in CT. In turn, RBM thickness was related to the BAL periostin and blood neutrophilia, albeit not to the systemic type-2 biomarkers. Therefore, we might speculate that separate mechanisms may drive airway remodeling, depending on whether they act at the bronchial mucosa level or airway wall structure. However, type-2 inflammation in the airways is likely involved in both of them.

Another interesting finding of our study is that the RBM thickening did not depend on asthma duration. Therefore, one might speculate that RBM thickening occurs early during the disease, e.g., as a response to the ongoing airway inflammation, and does not progress further due to implemented anti-inflammatory treatment. Noteworthy, this observation mirrors the study performed by Payne et al. [51]. They demonstrated that RBM thickening is already present in young children (median age 13 years) to a similar extent to milder adult asthmatics. These authors did not find an association with patients’ age, asthma duration, or lung function, either. Furthermore, it has been demonstrated that even children younger than five years old with confirmed wheezing episodes may present a thicker RBM layer [52].

Epithelial cells are the primary source of BAL periostin, stimulating TGF-β release, which activates the underlying fibroblasts to produce ECM components [6]. Although TGF-β is also secreted by eosinophils [4], the positive relationship between RBM thickness and BAL periostin suggests that the bronchial epithelium may indeed orchestrate mucosal and submucosa changes in airway remodeling pathology. However, the biological role of the thicker RBM in asthma remains uncertain. It has been hypothesized that it constitutes a secondary barrier that compensates for the increased epithelial fragility and leakiness, thus decreasing the entry of harmful particles into the submucosa [6]. However, Rijt et al. [53], based on murine experiments, have shown contrary results, indicating that basement membrane remodeling might promote new sensitizations by causing persistent activation of dendritic cells. Therefore, therapeutic intervention to stabilize RBM thickness, e.g., kinase inhibitors, might be beneficial for severe asthma patients. However, to recommend such a therapeutic approach, the specificity of RBM thickening and its determinants need to be comprehensively investigated. 

In our study, analysis of epithelial cell transcriptome revealed several gene groups associated with RBM thickness. As expected, in patients with thicker RBM, we demonstrated a higher expression of genes related to ECM proteins, such as *LAMA3* or *USH2A*. They encode the laminin subunit α_3_ and usherin, respectively, proteins that are essential for the basement membrane structure and function, widely expressed in many organs, including the lungs [17,18]. Several epithelial-mesenchymal growth regulators may increase *LAMA3* expression, such as epidermal growth factor (EGF) [17]. In turn, usherin contains laminin EGF-like and many fibronectin motifs, and thus, might be upregulated by laminin [18]. Therefore, both proteins are likely involved in increased ECM deposition and could participate in airway remodeling pathology. In this context, the decrease in expression of *LTBP1* can also be essential. This gene encodes a protein that maintains TGF-β in the latent ECM bound form [54]. Therefore, its downregulation might contribute to the increased TGF-β activity, promoting ECM remodeling and fibrosis.

Interestingly, RBM thickness remained in a strong positive association with the higher expression of multiple genes encoding proteins involved in cell activation, proliferation, and growth or with an anti-apoptotic function. On the other hand, some genes strongly—but inversely—related to RBM thickness have opposite roles, i.e., either antiproliferative or pro-apoptotic properties. This observation suggests that the airway epithelial transcriptome linked with thicker RBM promotes cell proliferation and growth, further reinforced by the upregulated genes referring to the increased cell metabolism, protein synthesis, cargo transport, and nucleic acid processing.

Another important finding of our study is the lower expression of genes involved in the formation of tight cell junctions. This outcome is in line with other reports pointing to the disrupted airway epithelial barrier in asthma [7]. It is believed that cleaving tight junctions results from external factors, such as allergens, pathogens, air pollutants, and an intrinsic predisposition [7]. Our report suggests that increased permeability of the epithelium may also contribute to subepithelial changes and airway remodeling. However, it is worth noting that parallel to the decreased cadherin mRNA levels, we also documented higher expression of *PNN*, an E-cadherin transcription activator, likely serving as a compensatory mechanism [55].

An airway epithelial cell transcriptome profile associated with thicker RBM might also reflect epithelial-to-mesenchymal transition, a process whereby epithelial cells acquire characteristics of fibroblasts or mucosa cells [6]. During such a structural modification, epithelial cells lose cell–cell polarity and adhesion, to become migratory and develop mesenchymal features [5]. For example, this is reflected in our data by the increased expression of actin-related proteins, such as *TPM1*, *TPM2*, *MYO1B*, and *CORO2A*.

The next group of genes associated with a thicker RBM, which merits comment, contains those referred to as the oxidative stress response genes. Some of these genes were reported as upregulated, such as *HSP90AB1*, promoting cell adaptation to environmental changes [56]; *SCARA3*, the product of which depletes reactive oxygen species [57], and *DNMT3A*, encoding an enzyme involved in CpG or non-CpG motifs DNA methylation [58]. However, decreased expression of *CA3* [48] and *HPRT* [40], both induced by hypoxia-inducible factor 1α (HIF1-α), a transcription factor that adapts the cell during oxygen deprivation, might suggest an impaired adaptation to oxidative stress in airway remodeling pathology. Furthermore, downregulated *HPRT* might be related to the local overproduction of uric acid [40], likely stimulating cell proliferation and new blood vessel formation [41,42]. Increased neovascularization might also be related to upregulated *FGD5* [59]. Interestingly, bronchial epithelial cells from patients with a thicker RBM also showed a gene profile that promotes nerve regeneration and functioning; therefore, it seems that neural network development may also occur in asthma remodeling pathology.

Another intriguing association shown in our data refers to the significant positive relationship between RBM thickness and the mRNA level of the gene encoding a decarboxylase, which catalyzes γ-aminobutyric acid (GABA) production. It has been recently demonstrated that the airway epithelium is a predominant source of endogenous GABA, contributing to airway smooth muscle tone relaxation [27]. Higher *GAD1* expression in those with thicker RBM likely indicates susceptibility to increased GABA epithelial production, partially explaining the lack of association between RBM thickness and spirometry values. Interestingly, the higher expression of this gene in the airway transcriptome has also been documented in chronic obstructive pulmonary disease (COPD) patients [28].

The last important issue that merits comment, is the relationship between the RBM layer and the expression of genes contributing to the immune response. A thicker RBM is strongly determined by upregulated *STAP1*, encoding a protein abundant in the stimulated B cells [37] and, to a lesser extent, *TRBV11-1*, encoding the variable domain of the T cell receptor [60]. Interestingly, a thicker RBM was also positively associated with gene transcripts encoding the proteins involved in the antiviral response and other innate immunity elements, such as *AMBRA1*, involved in autophagy regulation [26], and *MUC1* protecting cells against bacterial, viral, or enzyme attack [61].

Our study has several limitations. First of all, we did not analyze the airway transcriptome in healthy controls for ethical reasons. Therefore, we cannot exclude that similar genes also might determine the RBM thickness in controls. Asthma patients enrolled in this study were relatively old and had mostly moderate to severe disease; thus, the findings may not represent younger subjects with milder disease. We investigated bronchial brush biopsy; therefore, we speculate that the outcomes mainly refer to the airway epithelium and to a lesser extent, lung infiltrating inflammatory cells. We analyzed airway transcription profiles at a single time point; therefore, we cannot exclude their changes in time. Statistical associations reported here may not necessarily indicate cause–effect relationships. Finally, the clinical relevance of the demonstrated associations in the airway transcriptional profile in terms of disease severity, progression, and relation to airway remodeling requires further investigation.

## 4. Materials and Methods

### 4.1. Patients

Our patient group consists of 40 non-smoking moderate to severe asthma subjects aged 30–65 years, with at least a 3-year history of confirmed asthma and no exacerbation within six months before the enrollment. About half of the patients (*n* = 21) were characterized by persistent airflow limitation; the remaining had normal spirometry before or after the bronchodilator. Except for biological treatment, all asthma medications were permitted, including oral corticosteroids at a daily dose equivalent to ≤ 10 mg of prednisolone, unless the amount was the same during the preceding three months.

Diagnosis of asthma was established by a physician, based on recurrent respiratory symptoms (wheeze, cough, shortness of breath, and chest tightness), together with current or historically documented post-bronchodilator increase in FEV_1_ of at least 200 mL and 12% from the baseline [62]. Persistent airflow limitation was defined as a FEV_1_/VC index below 0.7 or FEV1 lower than 0.8 of predicted value after bronchodilator. Spirometry and bronchial reversibility test were performed according to the American Thoracic Society standards [63], using a Jaeger MasterLab spirometer (Jaeger-Toennies GmbH; Hochberg, Germany).

Ex-smokers were eligible for the study if they stopped smoking at least five years ago, with a history of fewer than seven pack-years. Arterial hypertension, diabetes mellitus, and hypercholesterolemia were allowed in study participants.

Definitions of asthma severity and co-morbidities, as well as other exclusion criteria, have been provided in Supplementary Materials.

The study was conducted following the Declaration of Helsinki, and the protocol was approved by the Ethics Committee of the Jagiellonian University (approval number: KBET/151/B/2013). All subjects gave written informed consent to participate in the study.

### 4.2. Lung CT

Lung CT with an assessment of airway cross-sectional geometry was performed one hour after 400 µg albuterol administration using 64-raw multidetector computed tomography (Aquilion TSX-101A, Toshiba Medical Systems Corporation, Otawara, Japan) in a helical scanning mode, without administration of intravenous contrast medium. Automated program AW Server (General Electric Healthcare, Wauwatosa, WI, USA) was used to quantify the airway cross-sectional geometry in the RB1 and RB10; including an average diameter of the lumen and airway, average wall thickness, lumen, and wall area, WAR and WTR, both defined in Supplementary Materials.

### 4.3. Bronchofiberoscopy, Endobronchial Biopsy, and Bronchial Brush Biopsy

Bronchofiberoscopy was performed according to the guidelines of the American Thoracic Society [64] using the bronchofiberoscope BF 1T180 (Olympus, Tokyo, Japan) with local anesthesia (2% lidocaine) and in mild sedation (0.05–0.1 mg fentanyl and 2.5–5 mg midazolam given intravenously). During that procedure, 2–3 endobronchial biopsies were taken from the right lower lobe (the carina between B9 and B10) together with the brush biopsy. Collected endobronchial specimens were immediately fixed in 10% neutral buffered formalin solution (Sigma-Aldrich, Saint Luis, MO, USA) and sent to the Pathology Department for further analysis. Brushes were immediately immersed in TRIzol Reagent (Thermo Fisher Scientific, Carlsbad, CA, USA).

### 4.4. Bronchoalveolar Lavage Fluid Analysis

The cytospin preparations (Thermo Scientific, Walthman, MA, USA) were made from bronchoalveolar lavage fluid (BALF) samples and stained with the May–Grunwald Giemsa dye. A total of 1000 cells in each preparation were counted; the results were shown as a percentage of all inflammatory cells (with the exception of epithelial cells). The remaining BALF sample was centrifuged; the supernatant was frozen in aliquots and stored at −70 °C until analysis.

### 4.5. Blood Laboratory Investigations

Fasting blood samples were drawn from the antecubital vein using minimal stasis between 8:00 and 11:00 A.M. Complete blood cell and platelet count and fibrinogen were assayed by routine laboratory techniques. High-sensitivity C-reactive protein and IgE were measured by latex nephelometry (Siemens, Marburg, Germany). Blood samples were drawn into serum tubes and centrifuged, similarly to BALF at 2000× *g* for 20 min. The supernatant was frozen in aliquots and stored at −70 °C until analysis.

Commercially available high sensitivity immunoenzymatic assays (ELISAs) were used to measure blood and BAL concentrations of IL-6, IL-10 (eBiosciencea, Vienna, Austria, both), and periostin (Phoenix Pharmaceuticals, Burlingame, CA, USA) and serum levels of a disintegrin and metalloproteinase domain-containing protein (ADAM)33 (Cloud-Clone Corp., Katy, TX, USA).

### 4.6. Histologic Examination

Tissue specimens were processed routinely, as described in our previous publication [65]—briefly, the 2 µm paraffin-embedded sections were cut and stained by hematoxylin and eosin. The slides were photographed by a Nikon D5300 camera attached to the Zeiss Axioscope microscope with a 100× oil immersion lens. The images were analyzed by the AnalySIS 3.2 software (Soft Imaging System GmbH, Germany). The RBM thickness was measured along the biopsy’s epithelial surface (Figure 2) according to the orthogonal intercept method suggested by Ferrando et al. [66] using arbitrary-distance units. We only analyzed sections covered by non-tangentially cut and well-preserved epithelial cells. For each patient, at least 30 individual RBM measurements were evaluated at intervals of 9.5 µm. The results were expressed as harmonic mean, defined in our previous publication [65].

### 4.7. RNA Preparation for Microarray Analysis

Total RNA was extracted from bronchial brush biopsy samples with TRIzol (Thermo Fisher Scientific, Carlsbad, CA, USA), fractioned by gravity gradient, and isolated using chromatographic columns (A&A Biotechnology, Gdynia, Poland), followed by storage in 96% ethanol at −80 °C. The quality of each RNA sample was assessed using Qiagen QIAxel system (Qiagen, Hilden, Germany), and RNA integrity was verified by agarose gel electrophoresis. RNA was reverse-transcribed into the cDNA library using Syngen Universal Script Reverse Transcriptase (Syngen, Wroclaw, Poland). The product was purified by Syngen PCR ME Mini Kit (Syngen, Wroclaw, Poland) and fluorescently labeled and purified using Kreatech ULS Platinum Bright Red/Orange Kit (Kreatech, Amsterdam, Netherland). Hybridization of cDNA to the Human Genomic 49K Mi ReadyArray (HEEBO) (Microarray Inc., Huntsville, AL, USA) at 37 °C for 24 h was performed, and microarrays subsequently were washed.

### 4.8. Microarray Data Retrieval and Bioconductor

Microarrays were scanned with an InnoScan 900 Microarray Scanner, and hybridization signals were detected using Mapix software (v.6.0.1; Innopsys, Carbonne, France). Since the Innopsys Mapix software failed to perform microarray gridding correctly in some cases, we developed a custom gridding algorithm to refine the results. Moreover, visible artifacts and noise render the task challenging. Therefore, the method based on maximally stable extremal regions (MSER) was applied to coordinate positioning markers and establish the sub-grid lines [67]. Computed results were verified manually and corrected if required. For our study, overall gridding performance exceeded that obtained with the commercial software, ultimately resulting in expression profiles of 33,519 gene products. Microarray data preprocessing (background correction and normalization) was done by Limma R software using the established methods [68,69]. We analyzed genomic data with Bioconductor v.3.7. the software of the R environment v.3.5.0.

Biological processes, molecular functions, and cellular components gene ontology terms associated with genes which expression levels correlated significantly with RBM thickness were derived from GO Biological Process 2018 by Gene Ontology Consortium. Only terms with *p*-value < 0.05 were shown (Supplementary Materials Tables S4–S6).

Gene and gene product descriptions were prepared based on the Gene Cards (The Human Gene Database) (https://www.genecards.org/), Entrez Gene (http://www.ncbi.nlm.nih.gov/gene), literature data, and gene ontology reports.

Semi-automated analytics platforms Cytoscape 3.7.1 and NetworkAnalyst 3.0 were used to create an interactions network, where individual molecular entities formed the nodes and their interactions the edges.

### 4.9. Statistical Analysis

Statistical analysis was performed with Statistica TIBCO 13.3 and R (version 3.6.1) software.

We used the Shapiro–Wilk test to verify data distribution. Continuous variables, mostly non-normally distributed, were reported as a median with interquartile range or as a mean with standard deviation, as appropriate. They were compared by the Mann–Whitney U-test or unpaired t-test, respectively. Categorical variables were given as percentages and compared by χ2 test. Lung CT variables and RBM thickness, were Box–Cox transformed and a one-way covariance analysis (ANCOVA) was performed to adjust for potential confounders, including age, sex, and BMI. To evaluate the relationship between continuous variables, a Spearman rank correlation test or univariate linear regression model with adjustment for age, sex, and BMI (CT parameters and RBM thickness) were performed. To calculate odds ratio with 95% confidence interval (CI), the cut-off point of BAL periostin concentration was calculated based on the receiver operating characteristic (ROC) curve.

We analyzed the Pearson linear and the Spearman rank correlation coefficients to study relationships between expressions of all genes and RBM thickness. The significance levels were established as *p* < 0.001 and *p* < 0.05, for the Pearson and Spearman rank correlation coefficients, respectively. We pointed out two genes group. The first was composed of 28 genes characterized by a powerful impact on the RBM thickness (at least 36%) with the absolute value of the correlation coefficient 0.6 and more, or −0.6 and less. The second group consists of genes with the correlation coefficients 0.4 to 0.599 and −0.599 to −0.4. All data regarding gene analyses, such as correlation tests and gene expression comparisons (Mann–Whitney U-test), were adjusted using the Benjamini–Hochberg correction.

A cluster analysis was performed using the k-means clustering method, based on mRNA levels of genes correlated best with RBM thickness. We obtained three different clusters compared by the covariance analysis (ANCOVA), Kruskal–Wallis-test, or χ2 test, as appropriate.

Results that presented a *p*-value less than 0.05 were considered statistically significant unless otherwise stated in the text.

## 5. Conclusions

Our data showed that RBM thickening likely progresses independently of the parameters linked with airway geometry and functioning, such as the rate of the decrease in spirometry values and the thickening of the bronchial walls in CT scans. A thicker RBM was associated with the airway epithelial cell transcriptome promoting cell activation, proliferation, and growth. Increased RBM thickness was also linked with altered expression of genes likely involved in the structural remodeling of the airways, such as increased ECM depositions, promoting neovascularization, and nerve functioning. However, the clinical relevance of the presented relationships, in terms of asthma severity, progress, and airway remodeling of larger airways requires further observational and experimental investigations.

## Figures and Tables

**Table 1:** Demographics and clinical characteristics of subjects studied.

	No-Persistent Airflow Limitation, *n* = 19	Persistent Airflow Limitation, *n* = 21	*p*-Value
**Demographic data**			
Age, years	52 (42–63)	56 (53–68)	0.07
Male gender, *n* (%)	5 (26)	3 (14)	0.58
Height, m	1.62 ± 0.08	1.63 ± 0.09	0.8
Body mass index, kg/m^2^	28.2 (24.9–31.3)	25.7 (23.9–31.2)	0.26
Past smoking, *n* (%)	5 (26)	5 (24)	0.86
Pack-years of smoking, *n*	0 (0–0)	0 (0–0)	0.46
Living primarily in inner-city environments, *n* (%)	9 (47)	12 (57)	0.76
**Asthma-Related Variables**			
Atopy, *n* (%)	11 (58)	9 (43)	0.95
Asthma duration, years	11 (5–20)	8.5 (5.5–18)	0.75
Asthma severity (GINA):			
persistent moderate, *n* (%)	8 (42)	7 (33)	
persistent severe, *n* (%)	11 (58)	14 (67)	0.57
Asthma symptom control §:			
well-controlled asthma, *n* (%)	2 (11)	2 (10)	
not-well controlled asthma, *n* (%)	4 (21)	5 (24)	
very poorly controlled asthma, *n* (%)	13 (68)	14 (67)	0.97
Oral glucocorticosteroids use, *n* (%)	5 (26)	5 (24)	0.86
FEV_1_ before bronchodilator, L	2.58 ± 0.84	1.67 ± 0.8	<0.001
FEV_1_ before bronchodilator, % of the predicted value	101 (85.9–110)	68.05 (48.3–79)	<0.001
FEV_1_ after bronchodilator, L	2.61 ± 0.81	1.92 ± 0.89	0.02
FEV_1_ after bronchodilator, % of the predicted value	103.8 (93–114.5)	73.7 (61.6–88.2)	<0.001
Change in FEV_1_ after bronchodilator, L	0.16 (0.03–0.29)	0.23 (0.15–0.34)	0.23
FEV_1_/VC (before bronchodilator)	74.1 (71.6–76.3)	56.7 (49–61.1)	<0.001
FEV_1_/VC (after bronchodilator)	77.8 (72–79.7)	62.3 (54.7–67.8)	<0.001

Categorical variables are presented as numbers (percentages), continuous variables as median and interquartile range, or mean and standard deviation, as appropriate. Abbreviations: FEV_1_—forced expiratory volume in one second, VC—vital capacity, GINA—Global Initiative for Asthma, L-liter, *n*—number, §—asthma symptom control (assessed based on asthma control test results).

**Table 2:** Laboratory variables.

	No-Persistent Airflow Limitation, *n* = 19	Persistent Airflow Limitation, *n* = 21	*p*-Value
**Blood Count**			
Hemoglobin, g/dL	13.4 (12.9–13.9)	13.7 (12.6–14.4)	0.67
Red blood cells, 10^6^/µL	4.62 (4.3–4.84)	4.71 (4.17–5.16)	0.71
White blood cells, 10^3^/µL	5.88 (4.95–7.51)	7.45 (6.51–9.25)	0.008
Neutrophils, 10^3^/µL	3.5 (2.7–4.2)	3.54 (2.7–4.2)	0.73
Lymphocytes, 10^3^/µL	1.9 (1.5–2.4)	2.2 (1.97–2.6)	0.13
Monocytes, 10^3^/µL	0.51 (0.48–0.68)	0.68 (0.53–1.1)	0.02
Blood platelets, 10^3^/µL	218 (177–246)	224 (187–294)	0.26
**Asthma and Inflammatory Biomarkers in Peripheral Blood**			
Eosinophilia/µL	130 (70–290)	565 (380–1340)	<0.001
Immunoglobulin E, IU/mL	43 (22.7–133)	152 (32–586)	0.19
C-reactive protein, mg/L	4.24 (0.45–8.67)	6.71 (0.58–42)	0.9
Interleukin 6, pg/mL	0.58 (0.43–1.04)	1.12 (0.5–3.21)	0.08
Interleukin 10, pg/mL	0.44 (0.005–0.76)	0.67 (0.41–1.62)	0.05
Periostin, ng/mL	0.3 (0.27–0.36)	0.38 (0.31–0.51)	0.06
A disintegrin and metalloproteinase domain-containing protein 33, ng/mL	1.2 (0.2–2.29)	1.76 (1.26–2.77)	0.11
**Reticular Basement Membrane Thickness**			
The thickness of the reticular basement membrane of the bronchial mucosa, µm	5.96 (5–7.96)	6.2 (5.24–7.45)	0.9
**Bronchoalveolar Lavage Cellularity and Biomarkers**			
Macrophages, %	86.8 (74.5–93)	83 (56.3–91)	0.47
Lymphocytes, %	7 (4.5–15.5)	7 (3–10.5)	0.33
Neutrophils, %	3 (2–4.5)	4 (2–12.5)	0.34
Eosinophils, %	0.1 (0–1)	2.3 (0.5-7)	0.004
Periostin, ng/mL	0.85 (0.76–0.96)	0.81 (0.72–0.95)	0.6
Interleukin 6, pg/ml	0.99 (0.67–1.14)	0.2 (0.005–0.86)	0.01
Interleukin 10, pg/ml	0.01 (0.005–0.01)	0.01 (0.005–0.01)	0.81

Variables are presented as medians and interquartile range.

**Table 3:** Lung computed tomography airway remodeling indices.

	No-Persistent Airflow Limitation, *n* = 19	Persistent Airflow Limitation, *n* = 21	*p*-Value	*p*-Value (Adjusted)
**The Right Upper Lobe Apical Segmental Bronchus (RB1)**				
Lumen diameter, mm	3.95 (3.6–4.5)	4.05 (3.7–5.3)	0.58	0.23
Airway diameter, mm	7.4 (6.8–8.4)	8.3 (7.3–9.3)	0.13	0.76
Wall thickness, mm	1.8 (1.6–1.9)	1.95 (1.8–2.4)	0.02	0.16
Wall thickness ratio	23.75 (21.3–24.6)	24.75 (23.1–26.9)	0.07	0.01
Lumen area, mm^2^	12.3 (10–16)	12.8 (10.5–22)	0.65	0.2
Wall area, mm^2^	30.8 (25.9–39.3)	39.4 (31.1–48.1)	0.1	0.78
Wall area ratio	72.55 (67.3–75.1)	74.8 (71.3–78.7)	0.08	0.01
**The Right Lower Lobe Basal Posterior Bronchus (RB10)**				
Lumen diameter, mm	4.35 (3.7–4.7)	3.8 (3–4.5)	0.17	0.02
Airway diameter, mm	7.9 (6.3–8.5)	7.6 (6.2–8.4)	0.36	0.03
Wall thickness, mm	1.8 (1.6–1.9)	1.8 (1.5–1.9)	0.98	0.54
Wall thickness ratio	23.1 (19.8–24.9)	24 (22–26.4)	0.16	0.07
Lumen area, mm^2^	14 (8.3–17)	11.1 (7.3–16.2)	0.31	0.04
Wall area, mm^2^	33.6 (22.4–42.4)	31.7 (21.9–40.5	0.5	0.08
Wall area ratio	71.3 (63.6–74.8)	73.3 (68.5–78.5)	0.18	0.06

Variables are presented as medians and interquartile range.

**Table 4:** Epithelial cell transcriptome—genes for which expression remained in strong positive or negative associations with reticular basement membrane thickness (correlation coefficients ≥ 0.6 or ≤ −0.6).

Gene	Alias for Gene	Correlation Coefficient	Potential Linkage with Airway and Asthma Pathology
*MIS12*	MIS12 kinetochore complex component	0.745	Plays an essential role in chromosome segregation [9]. The higher expression suggests increased proliferation potency of airway epithelial cells.
*CDK20*	Cyclin-dependent kinase 20	0.727	The encoded kinase may activate airway epithelial cell growth and proliferation [8].
*TACC2*	Transforming acidic coiled-coil protein 2	0.608	A centrosome- and microtubule-interacting protein, involved in homologous recombination repair [22], attenuates DNA damage and cytotoxicity in immortalized human bronchial epithelial cells [23].
*NEK5*	NIMA related kinase 5	0.625	Regulates centrosome integrity and is involved in cell proliferation [24]. The higher expression suggests increased proliferation potency of airway epithelial cells.
*ORC5*	The origin recognition complex subunit 5	0.603	Promotes DNA replication [25]. The higher expression might indicate increased proliferation potency of airway epithelial cells.
*AMBRA1*	Autophagy and beclin 1 regulator 1	0.626	Regulates autophagy, cell survival, and proliferation [26]. A positive correlation with reticular basement membrane (RBM) thickness suggests that autophagy mechanisms are likely involved in airway remodeling pathology.
*GAD1*	Glutamate decarboxylase 1	0.696	An enzyme catalyzing gamma-aminobutyric acid (GABA) production. GABA may be produced by human epithelial cells, likely relaxing airway smooth muscle [27]. Higher expression of *GAD1* in airway transcriptome has also been documented in chronic obstructive pulmonary disease (COPD) patients [28].
*MYO1B*	Myosin I B	0.658	A motor protein related to the actin filament-based cell organization and movement [29]. Higher airway epithelial cell expression might reflect epithelial-to-mesenchymal transition, a structural modification, whereby epithelial cells lose cell–cell polarity and display mesenchymal features, such as migratory properties [5].
*CORO2A*	Coronin 2A	0.6	An actin-binding protein involved in cell cycle progression, signal transduction, cell motility, apoptosis, and gene expression [30]. Higher expression in airway epithelium may also reflect the epithelial-to-mesenchymal transition.
*TPM1*	Tropomyosin alpha-1 chain	0.603	An actin-binding protein involved in the contractile cell system [31]. The higher expression may also reflect the epithelial-to-mesenchymal transition.
*OTOF*	Otoferlin	0.612	A protein involved in the vesicular release. Pathological mutations in *OTOF* are related to deafness. Although its exact role in asthma pathology is unknown, it may be involved in airway mucus secretion [32].
*TPH1*	Tryptophan hydrolase 1	0.643	A hydroxylase that catalyzes the first rate-limiting step in the biosynthesis of serotonin [33], an important inflammatory mediator in asthma. Serotonin modulates the function of various inflammatory cells, such as mast cells, eosinophils, dendritic cells, and lung epithelium. Higher levels have been shown in asthma patients’ airways; however, its exact source in the lungs is unknown [34].
*RNF34*	Ring finger protein 34	0.683	A ring finger containing protein, involved in protein–protein and protein–DNA interactions. It likely protects epithelial cells from premature apoptosis [35].
*EEF2*	Eukaryotic translation elongation factor 2	0.658	An essential factor in cell protein biosynthesis. The higher expression suggests increased protein translation potency in asthma patients with thicker RBM [36].
*STAP1*	Signal transducing adaptor family member 1	0.609	Positively regulates gene expression and signal transduction. The exact role in asthma pathology is unknown; however, it is abundant in activated human B cells [37].
*OAS2*	2′-5′-oligoadenylate synthetase 2	0.703	An interferon-induced antiviral ribonuclease L that destabilizes double-stranded viral RNA. It plays a critical role in the cellular innate antiviral response. A positive correlation with RBM thickness suggests that viral infections, essential asthma exacerbation triggers, might also be involved in airway remodeling pathology. The encoded protein also regulates cell apoptosis, growth, differentiation, and gene expression [20].
*USH2A*	Usherin	0.601	An extracellular matrix protein, found in many tissues’ basement membrane, including the lungs [18].
*LAMA3*	Laminin subunit α_3_	0.615	A protein belonging to the laminin family. Laminins are essential for the formation and function of the basement membrane [17]. Higher airway expression has been linked with COPD and idiopathic pulmonary fibrosis patients [38].
*PCDHA3*	Protocadherin alpha 3	0.609	A neural cadherin-like cell adhesion protein. Its role in airways is unknown; however, it might be related to the neural network and synaptic organization in airway remodeling pathology [39].
*PDS5*	Cohesin associated factor B	−0.643	A protein that negatively regulates cell proliferation [10]. The lower expression suggests increased proliferation potency of airway epithelial cells.
*HPRT1*	Hypoxanthine phosphoribosyltransferase 1	−0.647	A transferase engaged in purine salvage pathway. Its deficiency might be associated with increased local uric acid production [40]. Soluble uric acid is an endogenous danger signal, which may stimulate neovascularization [41] and cell proliferation [42]. Furthermore, hypoxia-inducible factor 1 α (HIF1-α) strongly upregulates *HPRT1*; therefore, a lower expression of *HPRT1* might indicate a worse response to oxidative stress in airway epithelium [40].
*RNF13*	Ring finger protein 13	−0.636	A ring zinc finger containing protein, promoting cell apoptosis [43]. The lower expression might indicate the anti-apoptotic potency of airway epithelial cells.
*MRPL37*	Mitochondrial ribosomal protein L37	−0.66	A large subunit of mitochondrial ribosomal proteins, which catalyzes protein production within the mitochondrion. Recent investigations have uncovered this protein’s pro-apoptotic role [44,45]; therefore, its lower expression might indicate anti-apoptotic potency of airway epithelial cells.
*CACNA1G*	Calcium voltage-gated channel subunit α1 G	−0.615	A transmembrane voltage-dependent calcium channel promoting cell death. The decreased expression suggests lower apoptotic activity [46].
*PIEZO2*	Piezo type mechanosensitive ion channel component 2	−0.6	A stretch-gated ion channel that senses airway stretches [47]. Lower expression in those with a thicker RBM might indicate a weaker response to epithelial cells’ mechanical stimuli. Lower *PIEZO2* expression has been shown in COPD patients [28].
*CA3*	Carbonic anhydrase 3	−0.606	A carbonic anhydrase that facilitates the reversible hydrating of carbon dioxide. Lower expression in those with thicker RBM might indicate a possible weaker contraction ability or a worse response to oxidative stress in airway epithelium because *CA3* is upregulated by HIF1-α [48].
*KIF11*	Kinesin family member 11	−0.645	A motor cell protein involved i.a. in cilia beating and cell division. The lower expression might suggest impaired cilia function in those with a thicker RBM [49].
*C21orf58*	Chromosome 21 open reading frame 58	−0.629	Exact biological roles are unknown

## Data Availability

The data presented in this study are available on request from the corresponding author. The data are not publicly available due to patients’ origin.

## Supplementary Materials

This article involves an online Supplement https://www.mdpi.com/1422-0067/22/3/998/s1. Table S1: Epithelial cell transcriptome. Asthma patients were divided based on the median value of the reticular basement membrane (RBM) thickness as a cut-off point; genes with higher or lower expression in those with thicker RBM are presented, Table S2: Genes for which expression remained in weaker, albeit significant positive associations with reticular basement membrane thickness (correlation coefficients 0.4 to 0.599), Table S3: Genes for which expression remained in weaker, albeit significant negative associations with reticular basement membrane thickness (correlation coefficients −0.599 to −0.4), Table S4. Biological processes gene ontology terms associated with genes which expression level correlated significantly with reticular basement membrane thickness, Table S5. Molecular functions gene ontology terms associated with genes which expression levels correlated significantly with reticular basement membrane thickness, Table S6. Cellular components gene ontology terms associated with genes which expression levels correlated significantly with reticular basement membrane thickness.
