# Supplementary material for: Reticular Basement Membrane Thickness Is Associated with Growth- and Fibrosis-Promoting Airway Transcriptome Profile-Study in Asthma Patients
Source: Int J Mol Sci. 2021 Jan 20;22(3):998. doi: 10.3390/ijms22030998 (PMC7863966; doi:10.3390/ijms22030998)
Supplement: Supplementary file 1 [file ijms-22-00998-s001.pdf]

Reticular basement membrane thickness is associated with growth- and fibrosis-promoting airway transcriptome profile – study in asthma patients

Stanislawa Bazan-Socha[1]*, Sylwia Buregwa-Czuma[2], Bogdan Jakiela[1], Lech Zareba[2], Izabela Zawlik[3], Aleksander Myszka[3], Jerzy Soja[1], Krzysztof Okon[4], Jacek Zarychta[1,5], Paweł Koźlik[1], Sylwia Dziedzina[1], Agnieszka Padjas[1], Krzysztof Wojcik[1], Michał Kępski[2], Jan G. Bazan[2*]

[1] Department of Internal Medicine, Jagiellonian University Medical College, Krakow, Poland

[2] College of Natural Sciences, Institute of Computer Science, University of Rzeszów, Pigonia 1, 35-310 Rzeszów, Poland

[3] Centre for Innovative Research in Medical and Natural Sciences, Institute of Medical Sciences, Medical College, University of Rzeszów, Kopisto 2a, 35-959 Rzeszów, Poland

[4] Department of Pathology, Jagiellonian University Medical College, Krakow, Poland

[5] Pulmonary Hospital, Zakopane, Poland

*- equal senior-author contribution

Supplementary Materials

Patients and Methods

Patients

The severity of asthma was categorized according to the Global Initiative for Asthma (GINA) guidelines. "Moderate" asthma was defined as mild persistent disease treated with a low dose of inhaled corticosteroids (ICS) (<250 µg of FP or equivalent) combined with long-acting β_2-agonists or a medium dose of ICS (250-500 µg of FP or equivalent). "Severe" asthma was defined as asthma that requires a high dose of ICS (>500 µg of FP or equivalent) with a long-acting β2-agonist to prevent it from becoming uncontrolled or asthma that remains uncontrolled despite this treatment.

The exclusion criteria included pregnancy or breastfeeding, any acute illness, congestive heart failure, coronary heart disease, atrial fibrillation, stroke, cancer, hyper- or hypothyroidism, liver injury, and chronic kidney disease (stage 3 or more) in a history.

Diabetes mellitus was defined as the current use of insulin or oral hypoglycemic medications or fasting serum glucose >7.0 mmol/l. Arterial hypertension was determined based on a history of hypertension (blood pressure >140/90 mmHg) or antihypertensive treatment administration. Hypercholesterolemia was defined as previously diagnosed and treated with statins, or serum total cholesterol >5.2 mmol/l. Liver injury was diagnosed if serum alanine aminotransferase was elevated (at least two times above the upper limit of the reference range). Coronary heart disease was defined as a documented history of coronary stenosis or stable angina.

Lung computed tomography

Wall thickness was calculated based on average outer and inner bronchial diameters. The wall area ratio (WAR) was defined as an average difference between the outer wall area and inner wall area divided by the outer wall area. The wall thickness ratio (WTR) was calculated as the average value of the ratio of the wall thickness and the airway diameter.

Results

Gene alias and gene product's description has been provided based on the Gene Cards (The Human Gene Database) (https://www.genecards.org/) or Entrez Gene (http://www.ncbi.nlm.nih.gov/gene).

Gene ontology was derived from GO Biological Process 2018 by Gene Ontology Consortium.

Table S1. Epithelial cell transcriptome. Asthma patients were divided based on the median value of the reticular basement membrane (RBM) thickness as a cut-off point; genes with higher or lower expression in those with thicker RBM are presented.

	**Gene**	**Alias for gene**	**Fold difference**	**p-value (adjusted)**	**The biological role of the protein encoded**	**Selected references (PMIDs)**
**Higher mRNA expression in patients with increased reticular basement membrane thickness (RBM values above the median)**						
1.	*TPH1*	Tryptophan hydrolase 1	1.209	0.0002	It is an aromatic amino acid hydroxylase that catalyzes the first rate-limiting step in the biosynthesis of serotonin, a vital hormone neurotransmitter, and melatonin	12379098, 19526457, 18982004, 20043001,
2.	*ZNF594*	Zinc finger protein 594	1.206	0.0008	A transcription factor, a biological role not fully understood, may be involved in transcriptional regulation	11347906, 7865130
3.	*USH2A*	Usherin	1.178	0.001	It is a protein that contains laminin epidermal growth factor, a pentraxin domain, and many fibronectin type III motifs; found in the basement membrane of many tissues, including lungs	19683999, 18452394, 15325563, 14970843, 10729113
4.	*OAS2*	2'-5'-oligoadenylate synthetase 2	1.168	0.0002	It is an interferon-induced antiviral enzyme that mainly recognizes virally produced double-stranded RNA and initiate RNA destabilization through activation of ribonuclease L; it may also mediate other cellular processes such as apoptosis, cell growth, differentiation, and gene expression	11682059, 21142819, 10464285, 9880569, 10464285
5.	*IPO13*	Importin 13	1.145	0.001	Functions in nuclear protein import as nuclear transport receptor; serves as a receptor for nuclear localization signals in cargo substrates	11447110, 19619331, 21139563, 20122403
6.	*SLC16A6*	Solute carrier family 16 member 6	1.134	0.0007	Catalyzes the rapid transport across the plasma membrane of many monocarboxylates such as lactate, pyruvate, branched-chain oxo acids derived from leucine, valine, and isoleucine, and the ketone bodies acetoacetate, beta-hydroxybutyrate, and acetate	16604139, 12739169, 16174808
7.	*CDK20*	Cyclin-dependent kinase 20	1.130	0.001	A kinase domain related to the cyclin-dependent protein kinases that may activate cyclin-dependent kinase 2 involved in cell growth and proliferation; it also controls the primary cilium structure by coordinating the ciliary membrane and axoneme	14597612, 21986944, 19672860, 19884882, 16954377
8.	*CLEC9A*	C-type lectin domain containing 9A	1.125	0.0002	Functions as an endocytic receptor on a small subset of myeloid cells, including dendritic cells, specialized for the uptake and processing of material from dead cells; it recognizes a	22483802, 18497879, 18408006, 12975309,

					filamentous form of actin in association with particular actin-binding domains of cytoskeletal proteins, including spectrin, exposed when cell membranes are damaged	21873635, 21677141, 18669894
9.	*HTRA2*	HtrA Serine Peptidase 2	1.124	0.001	Serine protease that promotes or induces cell death either by direct binding to and inhibition of BIRC proteins (also called inhibitor of apoptosis proteins), leading to an increase in caspase activity or by a BIRC inhibition-independent, caspase-independent, and serine protease activity-dependent mechanism (pro-apoptotic function)	10644717, 11583623, 10971580
10.	*MIS12*	MIS12 kinetochore complex component	1.2	0.0002	It is a part of the MIS12 complex required for normal chromosome alignment and segregation and kinetochore formation during mitosis	12515822, 15502821, 16585270, 23891108
11.	*LAMA3*	Laminin subunit α_3	1.199	0.0008	Is a protein belonging to the laminin family of secreted molecules; α_3 is a subunit of laminin-5, laminin-6, and laminin-7 of ECM proteins, essential for the formation and function of the basement membrane and regulating cell migration and mechanical signal transduction	8077230, 12915477, 12915477, 7633458
12.	*RNF157*	Ring Finger Protein 157	1.190	0.0009	It prevents apoptosis and promotes the survival of neurons; is also required for dendrite growth and maintenance	25342469, 25342469, 25342469
**Lower mRNA expression in patients with increased reticular basement membrane thickness (RBM values above the median)**						
1.	*BRINP2*	BMP/retinoic acid-inducible neural-specific 2	-1.486	0.001	Inhibits neuronal cell proliferation by negative regulation of the cell cycle transition	15193423, 14702039
2.	*PDS5B*	Cohesin associated factor B	-1.152	0.0008	It is a protein that interacts with the cohesin complex that holds together sister chromatids and facilitates accurate chromosome segregation during mitosis and meiosis; a negative regulator of cell proliferation and may be a tumor-suppressor gene	24141881, 15855230, 15489334, 29568061, 30948266
3.	*C21orf58*	Chromosome 21 open reading frame 58	-1.131	0.0002	Exact biological roles are unknown	10830953, 25416956
4.	*SLC39A13*	Solute Carrier Family 39 Member 13	-1.112	0.0005	A transmembrane protein functions as a zinc transporter	18513683, 21917916
5.	*PMP22*	Peripheral myelin protein 22	-1.11	0.0008	An integral membrane protein that is a major component of myelin in the peripheral nervous system; might be involved in growth regulation and myelinization in the peripheral nervous system	10212299, 8510709, 8252046, 19259128, 11456309

Table S2. Genes for which expression remained in weaker, albeit significant positive associations with reticular basement membrane thickness (correlation coefficients 0.4 to 0.599).

**Gene**	**Alias for gene**	**correlation coefficient**	**The biological role of the protein encoded**
RIT1	Ras-Like Protein Expressed In Many Tissues	0.433	A member of a subfamily of Ras-related GTPases, involved in regulating p38 MAPK-dependent signaling cascades in cellular stress, cooperates with nerve growth factors to promote neuronal development and regeneration
CARS2	Cysteinyl-TRNA Synthetase 2, Mitochondrial	0.541	A member of the class I family of aminoacyl-tRNA synthetases, encoded by the nuclear genome and transported to the mitochondrion, is essential in protein biosynthesis and catalyzes the ligation of cysteine to tRNA molecules
ARID1B	AT-Rich Interaction Domain 1B	0.531	A component of the chromatin remodeling complex may play a role in cell-cycle activation
PRAMEF9	PRAME Family Member 9	0.432	A protein that negatively regulates the apoptotic process and cell differentiation; positive regulates cell proliferation
TPM2	Tropomyosin 2	0.43	A member of the actin filament binding family binds to actin filaments in muscle and non-muscle cells; in association with the troponin complex, it plays a central role in muscle contraction; in non-muscle cells is implicated in stabilizing cytoskeleton
SSX5	SSX Family Member 5	0.579	A protein that belongs to the family of highly homologous synovial sarcoma X (SSX) breakpoint proteins functioning as transcriptional repressors. They are also capable of eliciting spontaneous humoral and cellular immune responses
ZNF521	Zinc Finger Protein 521	0.464	A transcription factor acts as an activator or a repressor depending on the context, with recognized regulatory functions in hematopoietic; it may be involved in neural tissue development
ZNF594	Zinc Finger Protein 594	0.583	A transcription factor, a biological role not fully understood
ZNF644	Zinc Finger Protein 644	0.495	A transcription factor, a biological role not fully understood
ZNF136	Zinc Finger Protein 136	0.439	A transcription factor, a biological role not fully understood
ESD	Esterase D,<br>S-Formylglutathione Hydrolase	0.572	A serine hydrolase active toward numerous substrates; may be involved in recycling sialic acids and in the detoxification of formaldehyde
BIRC3	Baculoviral IAP Repeat Containing 3<br>Inhibitor Of Apoptosis Protein 1	0.525	A member of the inhibitors of the apoptosis protein family, it inhibits apoptosis by binding to tumor necrosis factor receptor-associated factors TRAF1 and TRAF2, as well as modulates inflammatory signaling and immunity
WDR33	WD Repeat Domain 33	0.576	A member of the WD repeat protein family, may facilitate the formation of heterotrimeric or multiprotein

			complexes, is involved in various cellular processes, including cell cycle progression, signal transduction, apoptosis, and gene regulation.
POMZP3	POM121 and ZP3 Fusion	0.484	Involved in fertilization, a biological role in other tissues is not fully understood
DLAT	Dihydrolipoamide S-Acetyltransferase	0.521	A component E2 of the multi-enzyme pyruvate dehydrogenase complex that resides in the inner mitochondrial membrane and catalyzes the conversion of pyruvate to acetyl coenzyme A, is involved in glucose metabolism
OR8K5	Olfactory Receptor Family 8 Subfamily K Member 5	0.541	The olfactory receptor protein
TRMT10A	TRNA Methyltransferase 10A	0.552	A protein that belongs to the tRNA methyltransferase family, involved in mitochondrial protein synthesis
TCEAL5	Transcription Elongation Factor A Like 5	0.436	A transcription factor, a biological role not fully understood
TCEA2	Transcription Elongation Factor A2	0.496	A transcription elongation factor responsible for appropriate RNA polymerase II function interacts with general transcription factor IIB
CNOT2	CCR4-NOT Transcription Complex Subunit 2	0.471	A subunit of the multi-component CCR4-NOT complex regulates mRNA synthesis and degradation and is also thought to be involved in mRNA splicing, transport, and localization. The encoded protein interacts with histone deacetylases and functions as a repressor of polymerase II transcription.
SCFD1	Sec1 Family Domain Containing 1	0.469	Involved in vesicular transport between the endoplasmic reticulum and the Golgi
COMMD6	COMM Domain Containing 6	0.485	This protein downregulates activation of NF-kappa-B, inhibits TNF-induced NFKB1 activation
KIF27	Kinesin Family Member 27	0.575	A protein member of the kinesin family, it plays an essential role in microtube motor activity and epithelial cilium movement.
C1QTNF3	C1q And TNF Related 3	0.557	A member of the adipokine family, It regulates food intake, inflammation, apoptosis, vascular function, and ischemic injury
SPRED1	Sprouty Related EVH1 Domain Containing 1	0.477	A member of the Sprouty family of proteins inhibits growth-factor-mediated activation of MAP kinase
HSP90AB1	Heat Shock Protein 90 Alpha Family Class B Member 1	0.472	A member of the heat shock protein 90 family, a molecular chaperone that promotes the maturation, structural maintenance, and proper regulation of specific target proteins involved in cell cycle control and signal transduction, promotes RNA transcription and cell proliferation.
MROH2B	Maestro Heat Like Repeat Family Member 2B	0.509	Involved in fertilization; in other tissues, a biological role is unknown
MUC1	Mucin 1, Cell Surface Associated	0.417	A membrane-bound protein that is a member of the mucin family; it is expressed on the apical surface of epithelial cells, plays a role in intracellular signaling, and protects the cell against bacteria, virus, and enzyme attack

ACP6	Acid Phosphatase 6, Lysophosphatidic	0.573	A member of the histidine acid phosphatase protein family, balancing lipid composition within the cell
TP53I3	Tumor Protein P53 Inducible Protein 3	0.406	A protein involved in cellular responses to oxidative stresses and irradiation. This gene is induced by the tumor suppressor p53 and is thought to be involved in p53-mediated cell death
CAMTA1	Calmodulin Binding Transcription Activator 1	0.573	A protein that is a transcription factor with an activation function
RALGPS1	Ral GEF With PH Domain And SH3 Binding Motif 1	0.568	A guanine nucleotide exchange factor (GEF) for the small GTPase RALA; may be involved in cytoskeletal organization
INVS	Inversin	0.584	A protein that is likely involved in kidney development and left-right axis determination; biological roles in other tissues are unknown
ANKS1B	Ankyrin Repeat And Sterile Alpha Motif Domain Containing 1B	0.503	This protein interacts with amyloid-beta precursor and may have a role in the normal development of neuronal cells and Alzheimer disease pathogenesis
ANKFY1	Ankyrin Repeat And FYVE Domain Containing 1	0.461	This protein belongs to a subgroup of double zinc finger proteins which may be involved in vesicle or protein transport
TSFM	Elongation Factor Ts, Mitochondrial	0.457	A mitochondrial translation elongation factor
SPANXD	SPANX Family Member D	0.479	Involved in fertilization; biological roles in other tissues are unknown
THAP5	THAP Domain Containing 5	0.545	A regulator of cell-cycle; can function as a transcriptional repressor
LRRFIP2	LRR Binding FLII Interacting Protein 2	0.535	A protein encoded by this gene, along with MYD88, binds to the cytosolic tail of toll-like receptor 4 (TLR4), which results in activation of nuclear factor-kappa B signaling. Also, this protein can downregulate the NLRP3 inflammasome by recruiting the caspase-1 inhibitor to the inflammasome complex.
OLMALINC	Oligodendrocyte Maturation-Associated Long Intergenic Non-Coding RNA	0.414	An RNA gene that belongs to the long non-coding RNA class with a regulatory function
OTOF	Otoferlin	0.591	A protein involved in vesicle membrane fusion
URB1	URB1 Ribosome Biogenesis Homolog	0.523	Immunomodulator of the inflammatory response
FGD5	RhoGEF And PH Domain Containing 5	0.524	A protein that promotes neovascularization; regulates actin cytoskeleton and cell shape
CXorf51	Chromosome X Open Reading Frame 51A	0.455	A biological role is unknown
C17orf75	Chromosome 17 Open Reading Frame 75	0.406	A biological role is unknown
MROH6	Maestro Heat Like Repeat Family Member 6	0.502	A biological role is unknown
MAP6	Microtubule Associated Protein 6	0.47	A calmodulin-binding and calmodulin-regulated protein that is involved in microtubule stabilization in many cell types
MAP4K3	Mitogen-Activated Protein Kinase Kinase Kinase Kinase 3	0.457	A member of the mitogen-activated protein kinases; may play a role in response to environmental stress

ERFE	Erythroferrone	0.448	The iron-regulatory hormone that acts as an erythroid regulator after hemorrhage; promotes increased iron absorption and mobilization from stores and lipid uptake
NBEAL1	Neurobeachin Like 1	0.476	A protein likely involved in the vesicle trafficking, membrane dynamics, receptor signaling, pre-mRNA processing, signal transduction, and cytoskeleton assembly
CRISP1	Cysteine Rich Secretory Protein 1	0.454	Involved in fertilization; biological roles in other tissues are unknown
TRPV3	Transient Receptor Potential Cation Channel Subfamily V Member 3	0.411	A protein that belongs to a family of nonselective cation channels that function in various processes, including temperature sensation and vasoregulation
PACRGL	Parkin Coregulated Like	0.522	A biological role is unknown
NAA16	N-Alpha-Acetyltransferase 16, NatA Auxiliary Subunit	0.57	A ribosome binding protein, negative regulation of apoptosis, positive regulation of transcription, protein stabilization
GRID2	Glutamate Ionotropic Receptor Delta Type Subunit 2	0.542	A protein receptor for L-glutamate which acts as an excitatory neurotransmitter at many synapses in the nervous system
CSDE1	Cold Shock Domain Containing E1	0.421	An RNA-binding protein which is required, i.a. for internal initiation of translation of human rhinovirus and RNA turnover
FBXL20	F-Box And Leucine Rich Repeat Protein 20	0.477	A substrate-recognition component of the SCF (SKP1-CUL1-F-box protein)-type ligase complex; plays a role in neural transmission
EXOSC10	Exosome Component 10	0.577	A protein that participates in a multitude of cellular RNA processing and degradation
MS4A6A	Membrane Spanning 4-Domains A6A	0.479	A member of the membrane-spanning 4A gene family; may be involved in signal transduction
USP13	Ubiquitin Specific Peptidase 13	0.527	A protein involved in various processes such as autophagy and endoplasmic reticulum-associated degradation
PML	PML Nuclear Body Scaffold	0.577	A protein member of the tripartite motif (TRIM) family functions via its association with PML-nuclear bodies (PML-NBs) in a wide range of critical cellular processes, including tumor suppression, transcriptional regulation, apoptosis, senescence, DNA damage response, and viral defense mechanisms
SCARA3	Scavenger Receptor Class A Member 3	0.533	A protein that depletes reactive oxygen species and plays an essential role in protecting cells from oxidative stress
ISG20	Interferon Stimulated Exonuclease Gene 20	0.478	A protein that exhibits antiviral activity against RNA viruses in an exonuclease-dependent manner
NEKS	NIMA Related Kinase 2	0.592	A protein kinase involved in controlling centrosome separation and bipolar spindle formation in mitotic cells and chromatin condensation in meiotic cells
TENT4B	Terminal Nucleotidyltransferase 4B	0.563	A terminal nucleotidyltransferase that catalyzes the transfer of ATP and GTP preferentially on RNA 3’ poly(A) tail stabilizing mRNA

NOP14-AS1	NOP14 Antisense RNA 1	0.435	An RNA gene that belongs to the long non-coding RNA class with a regulatory function
RBM27	RNA Binding Motif Protein 27	0.448	An RNA binding protein positively regulates RNA export from the nucleus
PNN	Pinin, Desmosome Associated Protein	0.543	A transcriptional activator binding to the E-box 1 core sequence of the E-cadherin promoter gene; involved in the establishment and maintenance of epithelial cell-cell adhesion
SENP6	SUMO Specific Peptidase 6	0.495	A protein involved in chromosome alignment and spindle assembly
ATP13A5	ATPase 13A5	0.426	A member of the P-type transport ATPases family which transports inorganic cations and other substrates across cell membranes
CLEC7A	C-Type Lectin Domain Containing 7A	0.419	A member of the C-type lectin that functions as pattern receptors specific for beta-1,3-linked and beta-1,6-linked glucans, such as cell wall constituents from pathogenic bacteria and fungi
MTO1	Mitochondrial TRNA Translation Optimization 1	0.532	A mitochondrial protein thought to be involved in mitochondrial tRNA modification
DACT3	Dishevelled Binding Antagonist Of Beta Catenin 3	0.587	May be involved in the regulation of intracellular signaling pathways during development
AHRR	Aryl-Hydrocarbon Receptor Repressor	0.536	This protein participates in the aryl hydrocarbon receptor (AhR) signaling cascade; is involved in the regulation of cell growth and differentiation
TRBV11-1	T Cell Receptor Beta Variable 11-1	0.504	The V region of the variable domain of T cell receptor beta chain that participates in the antigen recognition
HNRNPLL	Heterogeneous Nuclear Ribonucleoprotein L Like	0.454	An RNA-binding protein that functions as a regulator of alternative splicing for multiple targets, including activation-induced alternative splicing in T cells
AP4B1	Adaptor Related Protein Complex 4 Subunit Beta 1	0.523	A heterotetrameric adapter-like complex 4 that is involved in targeting proteins from the trans-Golgi network to the endosomal-lysosomal system
IL1RAPL2	Interleukin 1 Receptor Accessory Protein Like 2	0.584	A member of the interleukin 1 receptor family, similar to the interleukin 1 accessory proteins, and is most closely related to interleukin 1 receptor accessory protein-like 1 (IL1RAPL1)
APOBEC3F	Apolipoprotein B MRNA Editing Enzyme Catalytic Subunit 3F	0.5	A DNA deaminase (cytidine deaminase) which exhibits antiviral activity
CIP2A	Cell Proliferation Regulating Inhibitor Of Protein Phosphatase 2A	0.467	It promotes independent cell growth and tumor formation
SLC6A5	Solute Carrier Family 6 Member 5	0.569	An integral membrane glycoprotein responsible for the clearance of extracellular glycine during glycine-mediated neurotransmission
DMRT2	Doublesex And Mab-3 Related Transcription Factor 2	0.508	Transcriptional activator required for the initiation and/or maintenance of the proper organization of the sclerotome, dermomyotome, and myotome

Table S3. Genes for which expression remained in weaker, albeit significant negative associations with reticular basement membrane thickness (correlation coefficients -0.599 to -0.4).

**Gene**	**Alias for gene**	**correlation coefficient**	**The biological role of the protein coded**
SPR	Sepiapterin Reductase	-0.523	An aldo-keto reductase that catalyzes the biosynthesis of tetrahydrobiopterin, secondary involved in neurotransmitter production, such as serotonin and dopamine
FAM27E5	Family with Sequence Similarity E5	-0.43	An RNA gene that belongs to the long non-coding RNA class with a regulatory function
CRHR1	Corticotropin Releasing Hormone Receptor 1	-0.402	A G-protein coupled receptor that binds the corticotropin-releasing hormone family and regulates diverse physiological processes, including stress, reproduction, immune response, and obesity
ANO3	Anoctamin 3	-0.401	A membrane protein with unknown biological function seems to act as a potassium channel regulator, involved in endoplasmic reticulum-dependent calcium signaling.
CDH23	Cadherin Related 23	-0.511	A member of the cadherin superfamily, cell-cell adhesion glycoproteins
DNMT3A	DNA Methyltransferase 3 Alpha	-0.515	A DNA methyltransferase to function in CpG and non-CpG motif methylation as an epigenetic modification
CNNM3	Cyclin And CBS Domain Divalent Metal Cation Transport Mediator 3	-0.433	Human transmembrane protein, involved in ion transport
XPR1	Xenotropic And Polytropic Retrovirus Receptor 1	-0.579	A protein that plays a role in phosphate homeostasis
KCNQ3	Potassium Voltage-Gated Channel Subfamily Q Member 3	-0.587	A part of the slow-acting potassium channel, important in the regulation of neuronal excitability
PCDHGC4	Protocadherin Gamma Subfamily C4	-0.41	A calcium-dependent cell-adhesion protein that may be involved in the establishment and maintenance of specific neuronal connections
DDX39A	DExD-Box Helicase 39A	-0.584	A member of the DEAD box protein family, implicated in several cellular processes involving alteration of RNA, such as translation initiation, nuclear and mitochondrial splicing, and ribosome and spliceosome assembly
SHPRH	SNF2 Histone Linker PHD RING Helicase	-0.575	A ligase involved in DNA repair; also acts as a transcription factor, and helicase
IL1RAP	Interleukin 1 Receptor Accessory Protein	-0.539	A component of the interleukin 1 receptor complex; it initiates signaling events that result in the activation of interleukin 1-responsive genes
WWP1	WW Domain Containing E3 Ubiquitin Protein Ligase 1	-0.53	A protein that plays an essential role in the regulation of a variety of cellular functions such as protein degradation, transcription, and RNA splicing
CEP112	Centrosomal Protein 112	-0.554	A protein belonging to the cell division control protein 42 effector protein family, a component of the human centrosome
ZNF625	Zinc Finger Protein 625	-0.582	A transcriptional regulator, the exact biological role is unknown

GPBP1L1	GC-Rich Promoter Binding Protein 1 Like 1	-0.561	A transcriptional regulator, the exact biological role is unknown
OR2T3	Olfactory Receptor Family 2 Subfamily T Member 3	-0.548	The olfactory receptor protein
ZBTB40	Zinc Finger And BTB Domain Containing 40	-0.466	A transcriptional regulator, the exact biological role is unknown
DOK4	Docking Protein 4	-0.544	A DOK protein that provides a docking platform for the assembly of multimolecular signaling complexes; may regulate the immune response induced by T-cells
MRPS25	Mitochondrial Ribosomal Protein S25<br>28S Ribosomal Protein S25, Mitochondrial	-0.478	A 28S subunit protein of mitoribosomes (small ribosomal unit) with pro-apoptotic properties
LTBP1	Latent Transforming Growth Factor Beta Binding Protein 1	-0.543	A protein that belongs to the family of latent TGF-β binding proteins (LTBPs), it is a crucial regulator of TGF-β activity by maintaining it in a latent state in extracellular space
RNFT1	Ring Finger Protein, Transmembrane 1	-0.458	A ligase that acts in the endoplasmic reticulum degradation pathway
NECAP1	NECAP Endocytosis Associated 1	-0.48	A protein localizes to clathrin-coated vesicles, involved in endocytosis
NCALD	Neurocalcin Delta	-0.488	A protein that belongs to the neuronal calcium sensor family; is involved in vesicle-mediated transport
ANK3	Ankyrin 3	-0.525	An ankyrin that was initially found in the central and peripheral nervous systems; ankyrins are a family of proteins that link the integral membrane proteins to the underlying spectrin-actin cytoskeleton and play roles in cell motility, activation, proliferation, and contact
CLDND1	Claudin Domain Containing 1	-0.584	A membrane protein, component of the tight junctions
C3orf52<br>*TTMP*	Chromosome 3 Open Reading Frame 52 or<br>TPA Induced Trans-Membrane Protein	-0.527	A membrane protein, the exact biological role is unknown
ATM	ATM Serine/Threonine Kinase	-0.512	This protein and the closely related kinase ATR are master controllers of cell-cycle checkpoint signaling pathways required for cell response to DNA damage and genome stability
NLGN1	Neuroligin 1	-0.558	A member of a family of neuronal cell surface proteins involved in cell-cell-interactions; plays a role in synapse function and synaptic signal transmission

Table S4. Biological processes gene ontology terms associated with genes which expression level correlated significantly with reticular basement membrane thickness.
Gene ontology derived from GO Biological Process 2018 by Gene Ontology Consortium

**Biological process**	**Accession number**	**Genes**	**p-value**	**Z-score**	**combined log (p-value) *z-score**
Positive regulation of synapse assembly	GO:0051965	*NLGN1; GRID2; IL1RAP*	0.0008	-38.23	119.25
Negative regulation of the viral life cycle	GO:1903901	*ISG20; APOBEC3F; OAS2; PML*	0.0009	-21.73	66.62
DNA damage response, signal transduction by p53 class mediator resulting in cell cycle arrest	GO:0006977	*MUC1; CNOT2; ATM; PML*	0.0009	-21.37	64.98
Signal transduction involved in mitotic G1 DNA damage checkpoint	GO:0072431	*MUC1; CNOT2; ATM; PML*	0.0010	-21.04	63.40
Excitatory synapse assembly	GO:1904861	*NLGN1; GRID2*	0.0013	-82.83	238.86
regulation of synapse assembly	GO:0051963	*NLGN1; GRID2; IL1RAP*	0.0018	-28.40	77.76
Histone mRNA catabolic process	GO:0071044	*EXOSC10; ATM*	0.0025	-60.24	156.42
DNA damage response, signal transduction by p53 class mediator	GO:0030330	*MUC1; CNOT2; ATM; PML*	0.0026	-16.16	41.86
Positive regulation of cell cycle arrest	GO:0071158	*MUC1; CNOT2; ATM; PML*	0.0026	-16.16	41.86
Positive regulation of cellular component biogenesis	GO:0044089	*GRID2; NLGN1; ATM; IL1RAP*	0.0032	-15.23	38.04
Positive regulation of DNA damage response, signal transduction by p53 class mediator	GO:0043517	*SPRED1; ATM*	0.0041	-47.33	112.83
Positive regulation of calcium ion-dependent exocytosis	GO:0045956	*NLGN1; CACNA1G*	0.0041	-47.33	112.83
Negative regulation of viral genome replication	GO:0045071	*ISG20; APOBEC3F; OAS2*	0.0051	-19.88	45.62
Positive regulation of nervous system development	GO:0051962	*GRID2; NLGN1; IL1RAP*	0.0057	-19.11	42.95
Positive regulation of signal transduction by p53 class mediator	GO:1901798	*SPRED1; ATM*	0.0061	-38.98	86.35
Translational elongation	GO:0006414	*TSFM; MRPS25; MRPL37; EEF2*	0.0062	-12.62	27.85

Positive regulation of cellular component organization	GO:0051130	*GRID2; NLGN1; ATM; IL1RAP*	0.0064	-12.50	27.41
Regulation of DNA-templated transcription, elongation	GO:0032784	*TSFM; TCEA2*	0.0084	-33.13	68.78
Regulation of ruffle assembly	GO:1900027	*NLGN1; STAP1*	0.0092	-31.55	64.20
Regulation of viral genome replication	GO:0045069	*ISG20; APOBEC3F; OAS2*	0.0096	-15.78	31.81
Neuronal action potential	GO:0019228	*ANK3; CACNA1G*	0.0110	-28.81	56.40
Membrane assembly	GO:0071709	*NLGN1; ANK3*	0.0110	-28.81	56.40
Histone mRNA metabolic process	GO:0008334	*EXOSC10; ATM*	0.0120	-27.61	53.06
Regulation of DNA damage response, signal transduction by p53 class mediator	GO:0043516	*SPRED1; ATM*	0.0130	-26.50	50.03
Regulation of cell communication	GO:0010646	*GRID2; NLGN1; BIRC3*	0.0133	-14.00	26.26
tRNA methylation	GO:0030488	*TRMT10A; MTO1*	0.0140	-25.49	47.27
Mitotic nuclear division	GO:0140014	*MIS12; KIF11; BIRC3*	0.0149	-13.43	24.55
Sensory perception of mechanical stimulus	GO:0050954	*PIEZO2; CDH23; USH2A*	0.0171	-12.74	22.51
Positive regulation of telomere maintenance	GO:0032206	*ATM; PML*	0.0208	-20.71	34.85
Mitochondrial translational elongation	GO:0070125	*TSFM; MRPS25; MRPL37*	0.0228	-11.42	18.76
Actin-myosin filament sliding	GO:0033275	*TPM2; TPM1*	0.0286	-17.44	26.91
Muscle filament sliding	GO:0030049	*TPM2; TPM1*	0.0286	-17.44	26.91
Regulation of ATPase activity	GO:0043462	*TPM2; TPM1*	0.0315	-16.57	24.88
Calcium-dependent cell-cell adhesion via plasma membrane cell adhesion molecules	GO:0016339	*NLGN1; CDH23*	0.0330	-16.16	23.95
Nuclear-transcribed mRNA catabolic process	GO:0000956	*EXOSC10; CNOT2; CSDE1; ATM*	0.0333	-7.62	11.25
Regulation of protein modification process	GO:0031399	*SPRED1; PML*	0.0344	-15.78	23.08
Mitochondrial translation	GO:0032543	*TSFM; MRPS25; MRPL37*	0.0386	-9.29	13.12
Mitotic spindle assembly	GO:0090307	*KIF11; BIRC3*	0.0407	-14.40	20.03
Positive regulation of action potential	GO:0045760	*ANK3*	0.0410	-55.22	76.61
Positive regulation of nucleobase-containing compound transport	GO:0032241	*RBM27*	0.0410	-55.22	76.61

Purine ribonucleoside monophosphate catabolic process	GO:0009169	*HPRT1*	0.0410	-55.22	76.61
Nuclear polyadenylation-dependent ncRNA catabolic process	GO:0071046	*EXOSC10*	0.0410	-55.22	76.61
Negative regulation of transforming growth factor beta production	GO:0071635	*HSP90AB1*	0.0410	-55.22	76.61
Synaptic transmission, glycinergic	GO:0060012	*SLC6A5*	0.0410	-55.22	76.61
Positive regulation of microglial cell activation	GO:1903980	*STAP1*	0.0410	-55.22	76.61
N-terminal peptidyl-methionine acetylation	GO:0017196	*NAA16*	0.0410	-55.22	76.61
Prepulse inhibition	GO:0060134	*GRID2*	0.0410	-55.22	76.61
Regulation of phagocytosis, engulfment	GO:0060099	*STAP1*	0.0410	-55.22	76.61
Regulation of nuclease activity	GO:0032069	*OAS2*	0.0410	-55.22	76.61
Regulation of dopamine metabolic process	GO:0042053	*HPRT1*	0.0410	-55.22	76.61
Cellular triglyceride homeostasis	GO:0035356	*C1QTNF3*	0.0410	-55.22	76.61
Negative regulation of necroptotic process	GO:0060546	*BIRC3*	0.0410	-55.22	76.61
Indolalkylamine metabolic process	GO:0006586	*TPH1*	0.0410	-55.22	76.61
Nuclear polyadenylation-dependent rRNA catabolic process	GO:0071035	*EXOSC10*	0.0410	-55.22	76.61
Regulation of adiponectin secretion	GO:0070163	*C1QTNF3*	0.0410	-55.22	76.61
Positive regulation of sodium ion transmembrane transport	GO:1902307	*ANK3*	0.0410	-55.22	76.61
Positive regulation of clathrin-dependent endocytosis	GO:2000370	*NLGN1*	0.0410	-55.22	76.61
Cellular response to magnesium ion	GO:0071286	*ANK3*	0.0410	-55.22	76.61
Negative regulation of delayed rectifier potassium channel activity	GO:1902260	*ANK3*	0.0410	-55.22	76.61
Maintenance of protein location in extracellular region	GO:0071694	*LTBP1*	0.0410	-55.22	76.61

Negative regulation of dendritic spine development	GO:0061000	*NLGN1*	0.0410	-55.22	76.61
Equilibrioception	GO:0050957	*CDH23*	0.0410	-55.22	76.61
Peptidyl-serine autophosphorylation	GO:0036289	*ATM*	0.0410	-55.22	76.61
Negative regulation of interleukin-6 secretion	GO:1900165	*C1QTNF3*	0.0410	-55.22	76.61
Glutamate catabolic process	GO:0006538	*GAD1*	0.0410	-55.22	76.61
L-amino acid import	GO:0043092	*SLC6A5*	0.0410	-55.22	76.61
Inner ear receptor cell differentiation	GO:0060113	*USH2A*	0.0410	-55.22	76.61
Positive regulation of RNA export from nucleus	GO:0046833	*RBM27*	0.0410	-55.22	76.61
Heterophilic cell-cell adhesion via plasma membrane cell adhesion molecules	GO:0007157	*NLGN1; GRID2*	0.0423	-14.10	19.37
RNA methylation	GO:0001510	*TRMT10A; MTO1*	0.0456	-13.52	18.13
Positive regulation of response to DNA damage stimulus	GO:2001022	*SPRED1; ATM*	0.0456	-13.52	18.13
Phosphate ion transmembrane transport	GO:0035435	*XPR1*	0.0477	-47.33	62.56
Purine nucleotide biosynthetic process	GO:0006164	*HPRT1*	0.0477	-47.33	62.56
Cellular divalent inorganic anion homeostasis	GO:0072501	*XPR1*	0.0477	-47.33	62.56
AV node cell action potential	GO:0086016	*CACNA1G*	0.0477	-47.33	62.56
Regulation of monocyte chemotactic protein-1 production	GO:0071637	*C1QTNF3*	0.0477	-47.33	62.56
Cellular trivalent inorganic anion homeostasis	GO:0072502	*XPR1*	0.0477	-47.33	62.56
Regulation of pinocytosis	GO:0048548	*ANKFY1*	0.0477	-47.33	62.56
Regulation of ER to Golgi vesicle-mediated transport	GO:0060628	*SCFD1*	0.0477	-47.33	62.56
Melanocyte differentiation	GO:0030318	*USP13*	0.0477	-47.33	62.56
Regulation of establishment of protein localization	GO:0070201	*SCFD1*	0.0477	-47.33	62.56
Positive regulation of histone H4 acetylation	GO:0090240	*MUC1*	0.0477	-47.33	62.56
Sequestering of extracellular ligand from receptor	GO:0035581	*LTBP1*	0.0477	-47.33	62.56

Pteridine-containing compound biosynthetic process	GO:0042559	*SPR*	0.0477	-47.33	62.56
Negative regulation of telomere capping	GO:1904354	*ATM*	0.0477	-47.33	62.56
Nuclear body organization	GO:0030575	*PML*	0.0477	-47.33	62.56
SA node cell action potential	GO:0086015	*CACNA1G*	0.0477	-47.33	62.56
Negative regulation of dendrite morphogenesis	GO:0050774	*NLGN1*	0.0477	-47.33	62.56
Positive regulation of membrane depolarization	GO:1904181	*ANK3*	0.0477	-47.33	62.56
Regulation of microglial cell activation	GO:1903978	*STAP1*	0.0477	-47.33	62.56
Negative regulation of cell adhesion mediated by integrin	GO:0033629	*MUC1*	0.0477	-47.33	62.56
Negative regulation of necrotic cell death	GO:0060547	*BIRC3*	0.0477	-47.33	62.56
Chromatin-mediated maintenance of transcription	GO:0048096	*ARID1B*	0.0477	-47.33	62.56
Cellular phosphate ion homeostasis	GO:0030643	*XPR1*	0.0477	-47.33	62.56
Regulation of telomere maintenance via telomerase	GO:0032210	*EXOSC10; ATM*	0.0490	-12.99	17.02
Calcium ion transport	GO:0006816	*CDH23; TRPV3; CACNA1G*	0.0492	-8.42	11.02

Table S5. Molecular functions gene ontology terms associated with genes which expression levels correlated significantly with reticular basement membrane thickness.
Gene ontology derived from GO Biological Process 2018 by Gene Ontology Consortium

Molecular functions	Accession number	Genes	p-value	z-score	combined log(p-value) *z-score
3'-5'-exoribonuclease activity	GO:0000175	*ISG20; EXOSC10; CNOT2*	0.0013	-32.06	92.74
Ion channel activity	GO:0005216	*PIEZO2; TRPV3; NCALD; CACNA1G*	0.0040	-14.25	34.10
Actin filament binding	GO:0051015	*MYO1B; TPM2; TPM1; CORO2A*	0.0120	-10.43	20.06
Actin binding	GO:0003779	*MYO1B; TPM2; TPM1; CORO2A; NCALD*	0.0324	-6.52	9.71
Exoribonuclease activity, producing 5'-phosphomonoesters	GO:0016896	*ISG20; EXOSC10*	0.0130	-26.50	50.03
NADP binding	GO:0050661	*TP53I3; SPR*	0.0184	-22.09	38.34
Phosphatidylinositol phosphate binding	GO:1901981	*MYO1B; RNF34; ANKFY1*	0.0189	-12.27	21.15
ATP-dependent microtubule motor activity, plus-end-directed	GO:0008574	*KIF11; KIF27*	0.0195	-21.37	36.53
Motor activity	GO:0003774	*MYO1B; KIF11; KIF27*	0.0214	-11.69	19.51
Exonuclease activity	GO:0004527	*ISG20; EXOSC10*	0.0259	-18.41	29.21
3'-5' exonuclease activity	GO:0008408	*ISG20; EXOSC10*	0.0286	-17.44	26.91
Cation transmembrane transporter activity	GO:0008324	*PIEZO2; ATP13A5; CACNA1G*	0.0302	-10.25	15.58
RNA binding	GO:0003723	*RBM27; TSFM; TRMT10A; APOBEC3F; HSP90AB1; MRPL37; URB1; HNRNPLL; EEF2; MTO1; WDR33; PNN; DDX39A; EXOSC10; OAS2; CSDE1*	0.0315	-3.82	5.74
ATP binding	GO:0005524	*MYO1B; HSP90AB1; OAS2; CARS2; MAP4K3*	0.0329	-6.50	9.63

Histone methyltransferase binding	GO:1990226	*HSP90AB1*	0.0410	-55.22	76.61
Transforming growth factor beta-activated receptor activity	GO:0005024	*LTBP1*	0.0410	-55.22	76.61
Mechanically-gated ion channel activity	GO:0008381	*PIEZO2*	0.0410	-55.22	76.61
Exoribonuclease activity	GO:0004532	*EXOSC10*	0.0410	-55.22	76.61
U3 snoRNA binding	GO:0034511	*ISG20*	0.0410	-55.22	76.61
ATP-dependent microtubule motor activity	GO:1990939	*KIF11; KIF27*	0.0439	-13.80	18.73
Adenyl ribonucleotide binding	GO:0032559	*MYO1B; HSP90AB1; OAS2; CARS2; MAP4K3*	0.0455	-5.94	7.97
SUMO-specific protease activity	GO:0016929	*SENP6*	0.0477	-47.33	62.56
Glycine transmembrane transporter activity	GO:0015187	*SLC6A5*	0.0477	-47.33	62.56
U1 snRNA binding	GO:0030619	*ISG20*	0.0477	-47.33	62.56
single-stranded DNA exodeoxyribonuclease activity	GO:0008297	*ISG20*	0.0477	-47.33	62.56
ubiquitin protein ligase binding	GO:0031625	*USP13; SHPRH; RNF34; AMBRA1; PML*	0.0484	-5.83	7.67

Table S6. Cellular components gene ontology terms associated with genes which expression levels correlated significantly with reticular basement membrane thickness.
Gene ontology derived from GO Biological Process 2018 by Gene Ontology Consortium

**Cellular component**	**Accession number**	**Genes**	**p-value**	**z-score**	**combined log(p-value) *z-score**
Clathrin vesicle coat	(GO:0030125)	*NECAP1; NCALD*	0.0007	-110.44	347.98
Polymeric cytoskeletal fiber	(GO:0099513)	*PNN; MYO1B; TPM2; TPM1; MAP6; KIF11; KIF27*	0.0009	-10.49	31.91
Axon initial segment	(GO:0043194)	*KCNQ3; ANK3*	0.0017	-73.62	204.43
Node of Ranvier	(GO:0033268)	*KCNQ3; ANK3*	0.0030	-55.22	139.12
Actin filament	(GO:0005884)	*MYO1B; TPM2; TPM1*	0.0066	-18.07	39.38
Main axon	(GO:0044304)	*KCNQ3; ANK3*	0.0220	-20.08	33.28
Nuclear chromosome, telomeric region	(GO:0000784)	*ORC5; ATM; PML*	0.0386	-9.29	13.12
Clathrin coat	(GO:0030118)	*NECAP1*	0.0410	-55.22	76.61
